# Alternative Splicing: A Critical Regulator in Human Bone Biology and Tumor Progression

**DOI:** 10.34133/research.0977

**Published:** 2025-11-19

**Authors:** Li Cao, Yuxiang Hu, Ke Jia, Miguel A Ruiz-Cardozo, Ethan Chen, Jonathan Yang, Zengwu Shao, Brian Andrew Van Tine, Wei Wu

**Affiliations:** ^1^ Department of Orthopaedic, Union Hospital, Tongji Medical College, Huazhong University of Science and Technology, Wuhan 430022, People’s Republic of China.; ^2^ Division of Medical Oncology, Department of Medicine, Washington University School of Medicine, St. Louis, MO 63110, USA.

## Abstract

Alternative splicing (AS) is the central mechanism of transcriptional regulation and generates diverse splice variants that influence protein structure, function, and intracellular activity. AS plays critical roles in tissue differentiation, organ development, and disease progression. This review focuses on the pivotal roles of AS in bone biology, highlighting its regulatory effects on osteoblasts, osteoclasts, chondrocytes, bone matrix remodeling, and bone remodeling, as well as the involvement of AS-related RNA-binding proteins in these processes. We also emphasize bone-specific AS events and their physiological importance in skeletal development and maintenance. Furthermore, the pathological role of AS is emphasized in bone-related tumors such as osteosarcoma, Ewing sarcoma, and chondrosarcoma. This review also explores aberrant AS mechanisms in bone metastatic cancers, including prostate, bladder, and breast cancers, with an in-depth analysis of their roles in tumor progression and alterations in the bone microenvironment. This review provides a comprehensive perspective on how AS factors, signaling pathways, and mechanical stimulation collaboratively regulate bone cells under both physiological and pathological conditions, paving the way for identifying potential intervention strategies. The mechanisms of AS in other pathological bone conditions, such as osteoporosis, osteoarthritis, and hereditary bone disorders, are also summarized. The potential applications of targeting AS in the diagnosis and treatment of bone diseases are discussed, offering insights into the underlying mechanisms and clinical translational potential.

## Introduction

Alternative splicing (AS) is an important post-transcriptional regulatory mechanism that enables a single gene to produce multiple distinct RNA and protein isoforms, thereby expanding the diversity of gene expression in eukaryotes. AS was discovered in 1977, when studies on the mRNA of adenovirus first revealed that a single gene can generate multiple mRNA transcripts through different splicing patterns [1,2].

Generally, RNA splicing can be categorized into 4 categories: group I self-splicing, group II self-splicing, nuclear mRNA spliceosomal splicing, and nuclear transfer RNA (tRNA) enzymatic splicing [3–5]. Group I splicing mainly occurs in genes of eukaryotic organelles, such as mitochondria and chloroplasts, whereas group II splicing occurs in genes of fungal mitochondria and plant chloroplasts [3,4,6]. Nuclear tRNA enzymatic splicing is mediated by specific endonucleases and has been extensively studied in yeast, a classical model organism for this mechanism [7]. Nuclear mRNA spliceosomal splicing is the process by which the spliceosome removes introns from nuclear mRNA, encompassing both constitutive splicing and AS [8]. Researchers commonly use the term “constitutive splicing” to refer to the basic form of splicing in which all exons are joined together without selection. However, as cells undergo developmental transitions and encounter increasing demands for protein function, a single gene can produce different RNA variants through AS, thereby encoding diverse protein isoforms [5]. Approximately 95% of multi-exon genes in the human genome undergo AS [9].

Precursor mRNA (pre-mRNA) splicing in humans is precisely mediated by the spliceosome and involves accurate recognition of splice sites and branch points, followed by intron removal and exon ligation [10]. AS is regulated by cis-acting elements and trans-acting factors and plays a critical role in bone biology [11]. The principal cell types in bone tissue, including osteoblasts, osteoclasts, and chondrocytes, undergo differentiation and perform functions precisely regulated by AS [12]. In addition, key components of the bone matrix, such as collagen and fibronectin, are subject to AS regulation, which influences the structure and function of bone tissue. However, most existing studies on AS in bone biology remain fragmented, with limited integration across osteogenesis, bone resorption, and bone matrix metabolism. This gap may obscure the overall connections of AS in regulating bone formation, resorption, and matrix homeostasis. In this review, we systematically summarize recent advances in AS within bone biology, emphasizing novel mechanistic insights and potential translational value.

**Fig. 1.**
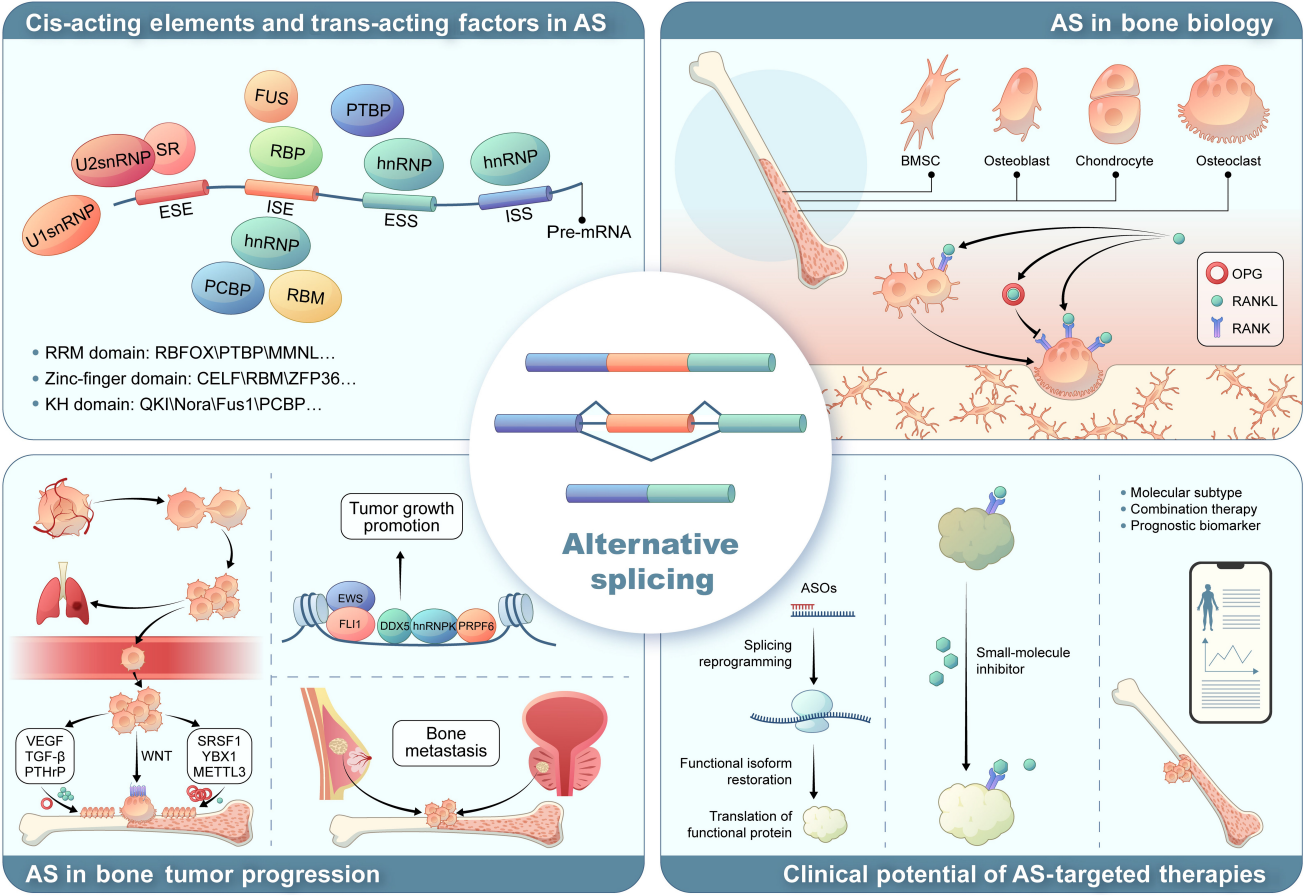
Overview of alternative splicing, including its cis-regulatory elements and trans-acting splicing factors, and its roles in bone biology, bone tumor progression, and the therapeutic potential of AS-targeted interventions. Specific sequence motifs on pre-mRNA and RNA-binding proteins with defined structural domains function as key regulators of alternative splicing. During bone development, alternative splicing modulates osteogenesis partly through regulation of the RANK signaling pathway. In bone tumors and bone metastases, dysregulated splicing plays a central role in disease progression. Therapeutic strategies targeting alternative splicing hold promise as a new avenue for treating bone-related and musculoskeletal disorders. AS, alternative splicing; ASO, antisense oligonucleotide; BMSC, bone marrow-derived mesenchymal stem cell; DDX5, DEAD-box helicase 5; DEAD-box RNA; ESS, exonic splicing silencer; ESE, exonic splicing enhancer; EWS, Ewing sarcoma breakpoint region 1; FLI1, friend leukemia integration 1; hnRNP, heterogeneous nuclear ribonucleoprotein; hnRNPK, heterogeneous nuclear ribonucleoprotein K; ISS, intronic splicing silencer; ISE, intronic splicing enhancer; METTL3, methyltransferase-like 3; OPG, osteoprotegerin; PCBP, poly(C)-binding protein; PRPF6, pre-mRNA-processing factor 6; PTBP, polypyrimidine tract-binding protein; RANK, receptor activator of NF-κB; RANKL, receptor activator of NF-κB ligand; RBP, RNA-binding protein; RBM, RNA-binding motif protein; RRM, RNA recognition motif; SRSF1, serine/arginine-rich splicing factor 1; SR, serine/arginine-rich protein; TGF-β, transforming growth factor β; U1snRNP, U1 small nuclear ribonucleoprotein; U2snRNP, U2 small nuclear ribonucleoprotein; VEGF, vascular endothelial growth factor; YBX1, Y-box binding protein 1.

Under pathological conditions, aberrant AS patterns are closely associated with the development and progression of bone tumors [13]. AS plays a critical role in the invasion and metastasis of osteosarcoma (OS), Ewing sarcoma (EwS), chondrosarcoma (CS), and various metastatic bone cancers, such as those originating from the prostate, bladder, and breast [11]. AS not only regulates tumor cell proliferation, migration, and apoptosis, but also contributes to the remodeling of both the tumor microenvironment and bone metastatic niche. Thus, AS variants serve as potential biomarkers for the diagnosis, prognosis, and therapeutic targeting of bone tumors [14]. Elucidating the specific mechanisms of AS and its roles in bone biology and tumor progression will provide novel insights and therapeutic strategies for the diagnosis and treatment of bone-related diseases (Fig. 1). Our review systematically summarizes the latest advances in the field, covering the fundamental processes and regulatory factors of human pre-mRNA splicing; the roles of key AS regulators in bone biology and bone tumors; the functions of representative AS events in bone remodeling and tumor progression; the molecular mechanisms by which RNA-binding proteins (RBPs) regulate AS in bone biology and bone tumors; and the potential translational value of AS targets in clinical applications.

## Splicing Process and Regulation of Pre-mRNA in Humans

### General splicing process in humans

Despite the inherent complexity of AS in humans, this section provides an overview of the most recently characterized proteins and molecular steps involved in the splicing process. Primary transcripts are single-stranded RNA products synthesized by DNA transcription. After processing, various mature RNA products, such as messenger RNA (mRNA), tRNA, and ribosomal RNA (rRNA), are produced. The pre-mRNA is a primary transcript, which is transcribed and synthesized by the DNA template in the nucleus, and mRNA is formed through the processes of 5′-cap addition, splicing, editing, and polyadenylation. Untreated pre-mRNA in the nucleus, collectively known as heterogeneous nuclear RNA (hnRNA), forms a heterogeneous nuclear ribonucleoprotein (hnRNP) after binding to a protein. Complexes of small RNA, nucleoproteins, and their attached introns are known as spliceosomes [10]. Noncoding small nuclear RNAs (snRNAs) bind to proteins to form small nuclear RNA protein particles (snRNPs), which play a precise splicing function. snRNAs (U1, U2, U4, U5, and U6 snRNAs) provide specificity for different types of splicing and identify various types of introns by base pairing (Table 1).

**Table 1.** Spliceosome primary structure and function in humans

Name	Function in splicing	Binding site	Component	Reference
U1 snRNP	U1 SnRNP recognizes the 5′-GU splicing sequence of introns, forms an early spliceosome complex (E-complex), and initiates splice assembly.	The intron nucleotides (GURAGN^a^) at the beginning of GU at the 5′ SS terminal of U1snRNA paired with C8 and A7 interact with each other by hydrogen bond and electrostatic interaction. U1-C forms a hydrogen bond with the sugar phosphate skeleton atom, which does not contact the RNA base but can improve the fault tolerance rate of U1snRNA pairing.	RNA: U1-snRNA Core protein: U1-70K, U1-A, U1-C and 7 core Smith (Sm) proteins	[10,369,370]
U2 snRNP	U2 snRNP could catalyze U6snRNA interaction, identify intron branching site (BS) sequence, recruit protein subunits, and form an A complex with U1 snRNP.	U2 snRNA binds the 30–40 nucleotide BS sequence (YNYURAY) before the intron boundary of 3′ SS.	RNA: U2-snRNA Core protein: SF3a (including SF3a60, SF3a66, and SF3a120 3 subunits) and SF3b (including SF3b155, SF3b130, SF3b145, SF3b49, SF3b14b, SF3b10, and SF3b14a 7 subunits), 3′ binding proteins (including 7 core Sm proteins, SNRPA1 and SNRPB2 3 subunits) and retention and splicing (RES) complex (including RBMX2, BUD13, and SNIP1 3 subunits)	[371,372]
U4 snRNP	U4 snRNP is part of the U4/U6.U5 tri-snRNP complex. U4 snRNP acts as a regulator of the U6 snRNA to maintain U6 in a precatalytic conformation.	U4 snRNA is base-paired by 24 highly conserved base pairs to U6 via stems I and II.	RNA: U4 snRNA Core proteins: 7 core Sm proteins and PRPF31	[373,374]
U5 snRNP	U5 snRNP is part of the U4/U6.U5 tri-snRNP complex. The 5′ SS and 3′ SS exons can bind to U5 snRNP, which helps the spliceosome strap the 5′ SS exon intermediate made in the first transesterification step and align it up with the 3′ SS exon for the second catalytic step via U5 snRNA loop I.	U5 snRNA loop 1 forms 4–5 base pairs with the 5′-exon during the reactions, which is important during exon ligation of pre-mRNA.	RNA: U5 snRNA Core proteins: 7 core Sm proteins. TXNL4A, SNRNP40, SNRNP200, CD2BP2, U5-52K, DDX23, PRPF28, PRPF6, PRPF8, and EFTUD2	[375,376]
U6 snRNP	U6 snRNP is part of the U4/U6.U5 tri-snRNP complex. The base pair specificity of U6 snRNA enables U6 snRNP to bind closely to U4 snRNA and loosely bind to U5 snRNA of U4/U6.U5 tri-snRNP at the initial stage of the splicing reaction. As the reaction progressed, U6 snRNA is decompressed from U4 and bound to U2 snRNA.	U6 snRNA is base-paired to the 5′ end of the intron during the splicing reaction before the lasso-shaped intermediate forms. Besides, the U6-U2 complex via base-pairing comprises the active site of the spliceosome.	RNA: U6 snRNA Core proteins: 7 core Sm proteins and SART3	[377–380]

^a^ Y, R, and N represent pyrimidine, purine, and nucleotide, respectively.

Intron 5′ splice site (5′ SS), branching point (BP) adenosine, and 3′ splice site (3′ SS) were identified during splicing [15]. In humans, nucleotide sequences flanking the 5′ SS and BP adenosine were not properly conserved, and the trinucleotide YAG sequence at the 3′ SS was preceded by a region of consecutive pyrimidine bases [16]. From a chemical perspective, the 2′ OH group of adenines located at the intron BP site initiates an attack on the phosphodiester bond, known as branching, at the intron 5′ SS. The 2′ oxygen of the first intron (G) at the 5′ SS was linked to BP adenosine, resulting in the formation of a lariat intron and the connection of the exons′ 3 OH groups [17]. Subsequently, the exon 3′ OH initiated an attack on the 5′-phosphate group of the first guanine base in the following exon, thereby substituting the 3′-terminal (known as exon ligation) of the lariat intron, ultimately leading to the fusion of the 2 exons. This process is known as constitutive splicing.

**Fig. 2.**
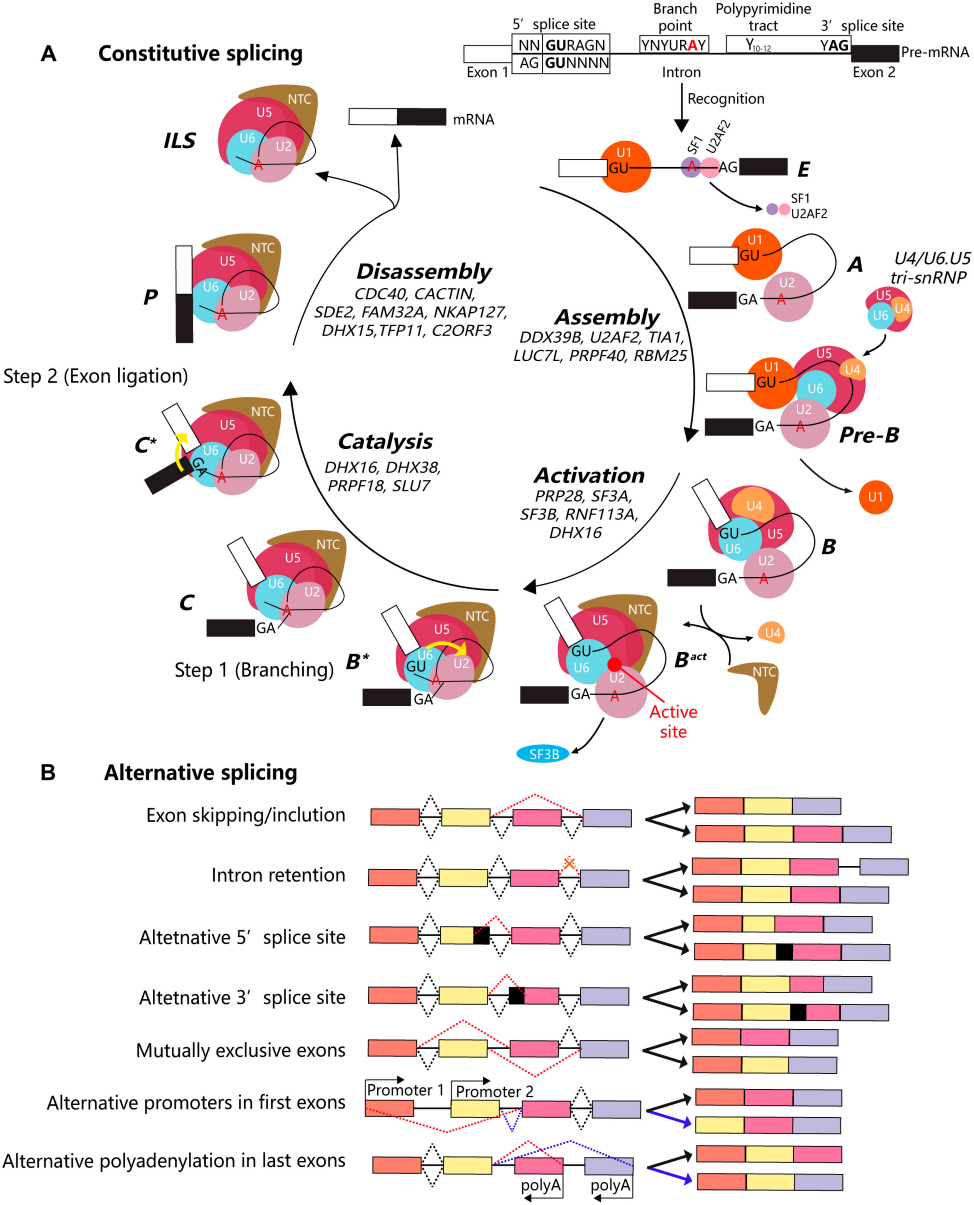
(A) Schematic representation of constitutive splicing. The figure illustrates the dynamic assembly of the spliceosome on pre-mRNA, including recognition of splice sites, formation of the pre-B and B complexes, activation of the catalytic core, branching at the intron lariat, exon ligation, and subsequent disassembly. Each stage is annotated with the major snRNPs and regulatory proteins involved, highlighting the stepwise mechanism that ensures accurate exon joining during mRNA maturation. (B) Overview of major alternative splicing patterns, including exon skipping/inclusion, intron retention, alternative 5′ and 3′ splice site usage, MXE, and regulation by APs or polyadenylation sites. CACTIN, cactin spliceosome associated protein; C2ORF3, chromosome 2 open reading frame 3; CDC40, cell division cycle 40; DDX16, DEAD-box helicase 16; DDX39B, DEAD-box helicase 39B; DHX15, DEAH-box helicase 15; DHX16, DEAH-box helicase 16; DHX38, DEAH-box helicase 38; FAM32A, family with sequence similarity 32 member A; TIA1, T-cell intracellular antigen 1; LUC7L, Luc7-like protein; NKAP127, NF-kappa-B activating protein 127; PRPF18, pre-mRNA processing factor 18; PRP28, pre-mRNA processing factor 28; PRPF40, pre-mRNA processing factor 40; RBM25, RNA binding motif protein 25; RNF113A, ring finger protein 113A; SF1, splicing factor 1; SF3A, splicing factor 3A; SF3B, splicing factor 3B; SDE2, SDE2 telomere maintenance homolog; SLU7, synthetic lethal with U5 snRNA 7 homolog; TFP11, TFP11 transcription factor-like protein; U2AF2, U2 small nuclear RNA auxiliary factor 2.

The spliceosome was not pre-assembled but rather constituted a sophisticated molecular machine (Fig. 2A). Currently, 10 different spliceosome states have been defined: E, A, pre-B, B, B^act^, B^*^, C, C^*^, P, and intron lariat spliceosome (ILS) complexes, and 4 distinct splicing phases: assembly, activation, catalysis, and disassembly. In addition to the 5 snRNPs, many splicing factors including RNA helicases and the nineteen complex (NTC) contribute to spliceosome function, comprising approximately 100 proteins. In the assembly phase, the U1 snRNP of the spliceosome recognizes the 5′-GU splicing sequence of the intron, and the zinc finger domain of U1-C touches the RNA duplex directly to stabilize the 5′ SS/U1 snRNA interaction. Human splicing factors, such as TIA1 (T-cell intracellular antigen 1), LUC7L (Luc7-like protein), PRPF40 (pre-mRNA processing factor 40), and RBM25 (RNA binding motif protein 25), are involved in promoting the binding of U1 snRNP to weak 5′ SS [18,19]. Afterwards, splicing factor 1 (SF1) is attached to the BP site of pre-mRNA, while the cofactor U2AF65-U2AF35 heterodimer acts together with the downstream polypyrimidine tract and 3′ SS to create the E complex. The DEAD (Asp-Glu-Ala-Asp)-box helicase replaces SF1 and U2AF (U2 small nuclear RNA auxiliary factor), recruiting U2 snRNP to the BP sequences to combine with U1 snRNP to form the lariat intron structure known as the A complex. The U4/U6.U5 tri-snRNP, the largest pre-assembled complex of spliceosome-containing active sites, was subsequently recruited to build the pre-B complex. U6 snRNA is paired with 5′ SS and folded to make a splice site. U4 snRNA maintained U6 in its pre-catalytic shape. U5 snRNA tethers 5′ SS exons together or aligns 5′ SS and 3′ SS during exon connection.

In the activation phase, PRP28 (pre-mRNA processing factor 28) helicase helps U1 snRNP break away from 5′ SS [20]. Subsequently, the U6 ACAGAGA loop is bound to 5′ SS to form the B complex. Structural alterations in the B complex facilitate the assembly of the U5-200kD helicase associated with U4 snRNP to disentangle the U4/U6 pair, resulting in the formation of splice active sites. After the U4 snRNP is released, U2 and U6 snRNAs pair to form the B^act^ complex [10]. The 4 U6 phosphates in the U2/U6 helix form a catalytic center. Under the stability of catalytic metal ions, NTC, and U2 core proteins, 5′ SS are incorporated into the catalytic center. However, SF3B in the B^act^ complex isolates U2 snRNP and removes the 2′ OH nucleophile of BP from the active site, whereas SF3A and RNF113A (ring finger protein 113A) are sequestered at 5′ SS. Upon entering the catalysis phase, the DHX16 (DEAH-box helicase 16) helicase remodels the B^act^ complex, enabling docking of the branch helix at the BP and 5′ SS, after which the active site catalyzes the branching reaction to form the B* complex. The C complex is formed when the phosphate group of the first intron is broken at 5′ SS (G+1 position) and connected to the 2′ OH of adenosine at the BP. The B* and C complexes are functionally similar, representing the spliceosome at the beginning (B*) and completion (C) of the branching step. Subsequently, DHX38 helicase restructures the C complex, leading to the transformation of the spliceosome into the C* complex before exon ligation [21]. 3′ SS is introduced into the active site of the C* complex, and with PRPF18 and synthetic lethal with U5 snRNA 7 homolog (SLU7), 5′ SS and BP enter the process of docking recognition. In the last stage of disassembly, the spliceosome forms a P complex under the action of exon ligation factors, such as CDC40 (cell division cycle 40), SLU7, CACTIN (coactivator of transcription 2), SDE2, FAM32A, and NKAP127 (nuclear karyopherin 127) [22–24]. At its active site, the 3′ SS, BP, and 5′ SS are aligned to allow the 3′ SS to initiate a nucleophilic attack on the exon 3′ OH of 5′ SS. After exon ligation, mRNA is released, and the P complex is transformed into the ILS. Under the influence of DHX15, TFP11, and C2ORF3, snRNP and NTC in the ILS are recycled and re-enter the splicing cycle.

Cryoelectron microscopy, cryoelectron tomography, and other methodologies have contributed to the rapid advancement of structural biology by gradually elucidating pre-mRNA splicing mechanisms [25]. Spliceosomes are composed of various proteins with an active core that functions as an RNA-based metalloenzyme [26]. This enzyme catalyzes 2 trans-esterification events: branching and exon ligation. The catalytic core remains constant during the transition from B to the B^act^ complex until mRNA release occurs. Furthermore, spliceosomes preserve both structural and mechanistic characteristics of yeast and humans. RNA helicases are crucial for facilitating core formation, regulating splice sites, and restructuring protein–RNA or RNA–RNA networks. The splicing process is further regulated by cis-acting elements and trans-acting factors, with both tissue specificity and disease specificity. This regulation results in 7 modes of AS of pre-mRNA: exon skipping (ES), intron retention (IR), alternative 5′ SS (A5SS), mutually exclusive exons (MXE), alternative 3′ SS (A3SS), alternative promoters (AP), and alternative polyadenylation (Fig. 2B). AS enhances gene expression complexity to adapt to the dynamic microenvironment, regulates cell growth and differentiation to determine cell fate, generates protein diversity without altering genomic integrity, improves coding efficiency by conserving genomic resources, and contributes to evolutionary innovation [27].

### AS process regulated by cis-acting elements

Theoretically, the cis-regulatory elements of pre-mRNA can be located anywhere within the transcript. However, most cis-acting elements are concentrated within 100 to 300 nucleotides of splice sites and exhibit distinct sequence characteristics [28]. Splicing enhancers are unique RNA motifs that influence AS by binding to splicing-related proteins. They can be classified into exonic splicing enhancers (ESEs) and intronic splicing enhancers (ISEs). The ESE motif is a purine-rich sequence of 3 to 8 nucleotides that is usually located near the end of the exon of the coding sequence. The ISE motif is typically 6 to 10 nucleotides rich in G and T, which can be categorized into 6 groups according to the features of the motif sequence; ISEs are often far away from exons (>500 nt) [29,30]. Previous studies have proposed that the SR family and SR-related splicing factors gathered on the ESE allow for both constitutive and AS, or the ability to select splice sites, by building complex networks of contacts between snRNPs [31,32]. For instance, an ESE mutation occurring in exon 18 of BRCA1 (breast cancer 1 gene) inhibits SF2 by identifying the splice site, thereby preventing the translation of mRNA in Clinical Genomics [33]. Mutation of ESE in adenomatous colonic polyposis leads to the loss of the exon splicing boundary of adenomatous polyposis coli, a suppressor gene resulting in the removal of exon 14 to produce a truncated protein that accelerates the development of familial adenomatous polyposis [34]. Unlike ESE, ISE functions as a binding site for RBPs, including hnRNPH1 (heterogeneous nuclear ribonucleoprotein H1), hnRNPF (heterogeneous nuclear ribonucleoprotein F), Fox-1 (feminizing locus on X homolog protein 1), and Fox-2, rather than for SR proteins [30,35]. In certain individuals, a 40-bp ISE was identified within the Alu repeat element of intron 20 of the ataxia-telangiectasia mutated (ATM) kinase gene, where it promotes the preservation of ATM exons through hnRNPA1 (heterogeneous nuclear ribonucleoprotein A1)/DAZAP1 (DAZ associated protein 1) binding [36,37]. A 19-bp ISE deficiency in intron 3 of proteolipid protein 1 (PLP1) in mice led to axonal damage and broad astrocyte proliferation, which is potentially associated with the hnRNPH/hnRNPF-mediated skipping of PLP1 exon 3.

Similarly, splicing silencers can be divided into exonic splicing silencer (ESS) and intronic splicing silencer (ISS). The ESS motif is rich in G and U and low in C, with a length between 4 and 10 nucleotides. The ISS motif exhibits a high abundance of A and U, and can be divided into 4 distinct groups varying in length from 3 to 5 nucleotides [30]. The general view is that the ESS and ISS limit the formation of snRNP assemblies around the spliced site by interacting with the hnRNP family [38]. An illustrative example is the ability of ESS to facilitate exon skipping in CD45 exons 4, 5, and 6. After the activation of T cells, hnRNPL (heterogeneous nuclear ribonucleoprotein L) interacts with the PSF (PTB-associated splicing factor) protein on the ESS, leading to the skipping of exon 4, 5, or 6, which creates a smaller CD45 variant that homodimerizes easily, accompanied by weak phosphatase activity [39,40]. Naturally occurring alterations in the ESS of CD45 contribute to increased susceptibility to autoimmune disorders and viral infections [41]. Furthermore, the presence of only 1 or 2 repeats of the ISS motif, especially GCACC or UGCACC, could effectively prevent exon splicing through YB1 binding in intron analysis for several genes [29]. Exon 7 skipping of survival motor neuron 2 (SMN2) pre-mRNA, a key gene in patients with spinal muscular atrophy (SMA), led to abnormal growth of motor neurons, whereas targeting 2 ISSs (UAGGGU and UAGGUC) in the proximal intron region of exon 7 flanks promoted the inclusion of exon 7 of SMN2 by inhibiting hnRNPA1/A2-mediated splicing, highlighting the therapeutic potential of ISS in diseases.

However, cis-acting elements in AS are worth reviewing. The mechanism of binding of the splicing factor to the RNA motif was characterized by adaptability, and the regulation of the RNA-protein network exhibited considerable plasticity in various clinical situations. In addition, a study of the RNA recognition motif of the SR and hnRNP protein families indicated that they exhibited antagonistic or cooperative influences on the cis-regulatory elements in AS and were susceptible to the influence of other splicing factors [5,31,42]. Although the characterization of cis elements has advanced, the methods for identifying cis-acting elements need to be improved. The predominant technique for identifying short splicing sequences is the cell-based fluorescence-activated screening approach; however, no studies have used histological identification methods [29,30,43].

### AS process regulated by trans-acting factors

Splicing is accompanied by profound conformational rearrangements and is regulated by cis-acting elements, trans-acting factors, and splicing factors. This intricate regulation hints at the existence of a “splicing code”. RBPs participate directly or indirectly in AS regulation, and their splicing-regulatory activity is primarily determined by the composition and specificity of the sequences they recognize. Among these, serine/arginine-rich (SR) proteins and hnRNPs are the best characterized.

Humans possess at least 12 distinct genes encoding SR proteins, distributed across various chromosomes [44,45]. SR proteins are characterized by an arginine/serine-rich (RS) domain and at least one RNA recognition motif (RRM) that ensures RNA interactions. Upon phosphorylation, SR proteins bind to ESE through their RS domains and promote exon inclusion by recruiting U1 snRNP and U2 snRNP. The greater the density of serine–arginine repeats within the ESE, the higher the relative activity of the SR proteins [46]. The proximity to introns further enhances their ability to promote exon inclusion. A subset of SR proteins can also bind to introns within pre-mRNAs to interfere with spliceosome assembly [47]. For instance, following adenoviral infection, serine/arginine-rich splicing factor 1 (SFRS1) binds intronic silencer elements in adenoviral pre-mRNA, preventing U2 snRNP binding to the 3′ SS [48].

hnRNPs form another classical protein family involved in AS regulation [49]. At least 19 genes encoding hnRNPs have been identified, each of which is capable of producing multiple variants [50]. Similar to SR proteins, hnRNP family members contain at least one RRM domain alongside auxiliary regions, such as RGG or KH motifs, to facilitate interactions with pre-mRNA and proteins. hnRNPs are generally considered negative regulators of AS. For example, hnRNP L suppresses AS by binding CA-rich sequences and interfering with U1 snRNP binding to the 5′ SS of the SLC2A2 pre-mRNA [51]. Similarly, hnRNP A1 inhibits the inclusion of variable exon N1 in c-Src and competes with SFRS1 for binding [52]. hnRNP A1 binds to the UAG sequence through its RRM1 and RRM2 domains, facilitating the skipping of exon 13 in lysyl oxidase like 2 (LOXL2), ultimately promoting the expression of a shorter LOXL2 variant [53].

Other RBPs involved in AS regulation were predominantly enriched in the RRM, Zinc-finger, and KH domains. Notably, 86% of RRMs bind to single-stranded mRNA [54]. Among 230 human RBPs containing RRMs, 29% were functionally annotated to be involved in AS [54]. Zinc-finger domains not only bind RNA to participate in AS but also interact with DNA and other proteins [55,56]. Furthermore, 57% of the zinc finger proteins exhibit AS dysregulation when knocked out in embryonic stem and neuroblastoma cells [57]. KH domains, first identified in hnRNPK (heterogeneous nuclear ribonucleoprotein K) homology, are composed of approximately 70 amino acids and exhibit high specificity and affinity to C-rich polypyrimidine motifs in introns near 5′ SS, facilitating exon splicing [58]. In addition to the hnRNP and SR families, other proteins with RRM domains that are involved in AS include RBFOX, PTBP, MBNL, and CELF. Proteins containing KH domains include QKI, KHDRBS1, Nova, FUS, SF1, and PCBP. Those with zinc finger domains include CELF, ZFP36, RBM, PRDM, ZNF277, ZNF768, ZFP36L1, ZFP36L2, WT1, ZMAT2, and TRIM28 (Table 2).

**Table 2.** Cis-acting elements and trans-acting factors in alternative splicing

	Name	Function
Cis-acting elements	Splice sites	5′ SS and 3′ SS.
		5′ SS binds to U1 snRNP and 3′ SS binds to U2 snRNP.
	BPS	5′-ynyuray-3′ (r = A/G, y = C/U, n = A/T/C/G).
		Bind to U2 snRNP.
	ESE	About 6 nucleotides in the exon. Bind to SR protein to guide or enhance the accurate splicing of hnRNA or pre-mRNA to mRNA.
	ESS	4–18 nucleotide sequence.
		Bind to hnRNPs to block exon splicing and inhibit intron retention.
	ISE	6–10 nucleotide sequence.
		Bind to SR protein to improve the activity of AS sites.
	ISS	6–10 nucleotide sequence.
		Bind to hnRNPs to inhibit the activity of AS sites.
Trans-acting splicing factors	hnRNP family	Negative regulatory factors.
		Bind to ESS or ISS, inhibit the recognition of nearby splice sites.
	SR family	Positive regulatory factors.
		Bind to ESE or ISE, promote the activity of adjacent splice sites.
	Other RBP	Proteins with RNA-binding motifs, such as RRM, Zinc-finger, or KH domains, are involved in regulating exon or intron AS. However, the regulatory functions of these RBPs are not limited to AS.

## Role of AS in Bone Biology

### Osteoblasts

**Fig. 3.**
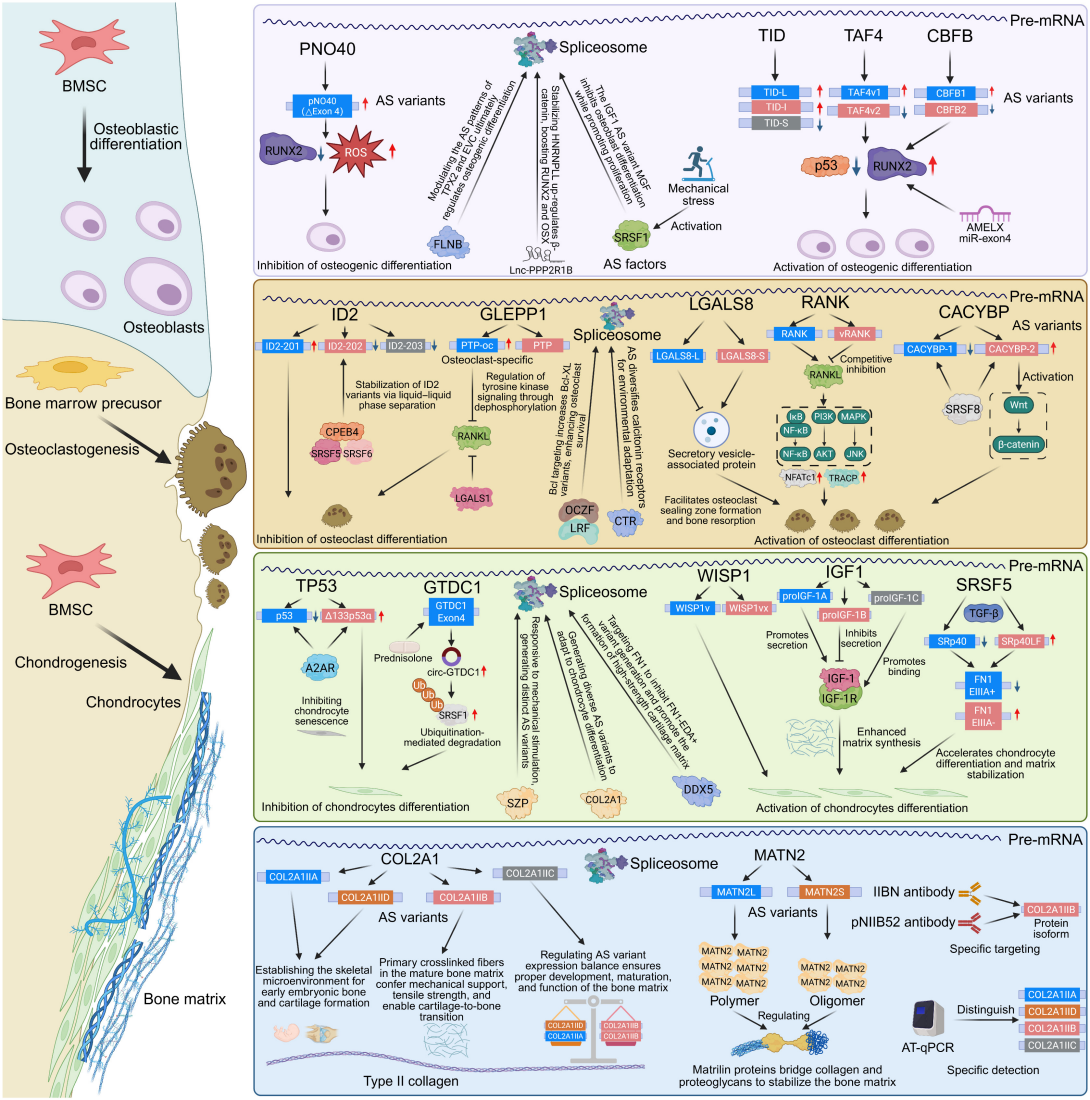
Alternative splicing landscape of bone biology. The schematic illustrates how AS events regulate different cellular compartments of the skeletal system. In osteoblasts, variants such as PNO40, TAF4, and CBFB, along with splicing factors like SRSF proteins, modulate RUNX2 activity, ROS signaling, and mechanical stress responses to control osteogenic differentiation. In osteoclasts, AS-regulated genes including ID2, RANK, CACYBP, and LGALS8 influence osteoclast differentiation and bone resorption through pathways involving RANKL signaling, tyrosine kinase activity, and vesicle secretion. In chondrocytes, aberrant splicing of TP53, GTDC1, WISP1, and IGF1, as well as altered expression of splicing regulators such as DDX5 and SRSF5, impacts senescence, matrix synthesis, and cartilage stabilization. Within the bone matrix, AS variants of structural proteins such as COL2A1 and MATN2 determine collagen composition, matrix organization, and mechanical integrity. CACYBP, calcyclin binding protein; CBFB, core-binding factor subunit beta; COL2A1, collagen type II alpha 1 chain; FLNB, filamin B; GLEPP1, glomerular epithelial protein 1; GTDC1, glycosyltransferase-like domain-containing protein 1; ID2, inhibitor of DNA binding 2; IGF1, insulin-like growth factor 1; LGALS8, galectin-8; MAML1, mastermind-like protein 1; MATN2, matrilin-2; PNO40, partner of NOL1 homolog; RANK, receptor activator of nuclear factor kappa-B; RUNX2, runt-related transcription factor 2; SRSF1, serine/arginine-rich splicing factor 1; SRSF5, serine/arginine-rich splicing factor 5; TAF4, TATA-box binding protein associated factor 4; TID, TID1 tumorous imaginal disc 1; TP53, tumor protein p53; WISP1, WNT1 inducible signaling pathway protein 1; p53, tumor protein p53.

We now turn to the roles of AS in the regulation of bone biology. Osteogenesis and chondrogenesis are critical physiological processes in bone biology in which AS plays a substantial role (Fig. 3). During the pubertal phase of skeletal development, osteoblasts become highly active, depositing new bone matrix along the periosteal surface and secreting osteocalcin and lipocalin to modulate systemic metabolism [59]. The prevailing view is that osteoblasts, key participants in osteogenesis, originate from bone marrow mesenchymal stem cells (BMSCs) [60]. The process by which stem cells differentiate into osteoblasts is called osteogenic differentiation, and numerous AS events contribute to this process. For instance, one study has identified 3 AS variants of the heat shock protein TID (encoded by DNAJA3): TID-L, TID-I, and TID-S. Among these, TID-L and TID-I variants were increased during the differentiation of BMSCs into osteoblasts, whereas TID-S was minimally expressed, suggesting its involvement in osteogenic differentiation [61]. Filamin B, a newly discovered RBP, regulates the AS of transcription factors Tpx2 and Evc, which are associated with early osteoblast development and thereby influence osteoblast differentiation [62]. In addition, long AS variants of PNO40 (partner of NOL1 homolog), a core protein of the 60S ribosome, potentially promote osteogenic differentiation and inhibit senescence by down-regulating runt-related transcription factor 2 (Runx2) and up-regulating reactive oxygen species (ROS) [63]. Another study has demonstrated that variant I of transcription factor IID subunit TATA-box binding protein associated factor 14 (TAF14) is abundantly expressed in human mesenchymal stem cells (hMSCs), whereas variant II is relatively rare. When the transcription activation factor homology (TAFH) domain encoded by TAF14 exons 5 and 7 is absent, hMSCs are driven toward chondrogenic differentiation, thereby blocking other differentiation pathways [64]. Compared with demethylases, the N6-methyladenosine (m6A) methyltransferase Mettl3 is up-regulated in BMSCs, and it regulates the expression of Vegfa and its AS variants, thereby promoting osteogenic differentiation [65].

Noncoding RNAs also modulate AS programs to regulate osteogenic differentiation. Specifically, a long non-coding RNA called lnc-protein phosphatase 2 regulatory subunit A beta (PPP2R1B) interacts with heterogeneous nuclear ribonucleoprotein L-like (HNRNPLL), leading to the down-regulation of variant 201 and the up-regulation of variant 203 of PPP2R1B. This results in the dephosphorylation and stabilization of β-catenin, facilitating its nuclear translocation to activate the Wnt (Wingless/Integrated)/β-catenin pathway and promote the expression of Runx2 and OSX (Osterix), ultimately enhancing osteogenesis [66]. Similarly, the co-transcription factor, CBFB (core-binding factor subunit beta), which interacts with Runx2, undergoes extensive AS in vivo, resulting in the predominance of the CBFB2 variant over the CBFB1 variant. This restricts osteogenic differentiation to appropriate levels, thereby benefiting skeletal development [67]. Variants of WNK lysine-deficient protein kinase 1 (Wnk1), collagen type IX alpha 1 chain (Col9a1), and cyclin-dependent kinase inhibitor 2a (CdkN2a) dominate different stages of osteoblast development, suggesting their potential role as key players in osteogenic differentiation [68]. Moreover, AS programs produce novel miRNAs that regulate osteogenic differentiation. For example, exon 4 of amelogenin (encoded by the *AMELX* gene) is spliced to form miR-exon4, which is proposed to promote osteoblast differentiation by regulating Runx2 expression [69].

Bone and cartilage formation cannot be separated from external mechanical stimulation, and current research indicates that AS is involved in the mechanical stimulation-induced formation of bone and cartilage. A previous study has demonstrated that mechanical stimulation activates the AS factor serine/arginine-rich splicing factor 1 (SRSF1) in osteoblasts, leading to the up-regulation of an insulin-like growth factor 1 (IGF1) splice variant named mechano growth factor (MGF). The MGF variant arises from the specific retention of exon 5 in IGF1 pre-mRNA, which alters the reading frame and causes premature termination of translation, resulting in a truncated protein with mechanosensitive capacity [70,71]. The MGF variant ultimately inhibits osteogenic differentiation via the extracellular signal-regulated kinase (ERK) signaling pathway while promoting osteoblast proliferation and migration [72]. Additionally, the AS factor SRSF1 responds to mechanical stimulation by co-regulating the AS of cyclin D1 with the SWI/SNF complex subunit SMARCE1 (SWI/SNF-related matrix-associated actin-dependent regulator of chromatin subfamily E member 1). This process reduces cyclin D1a levels and increases cyclin D1b levels [73].

**Fig. 4.**
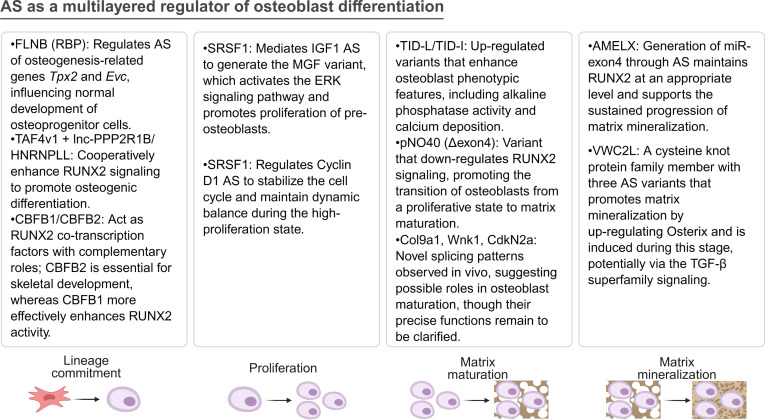
Alternative splicing acts as a multilayered regulator of osteoblast differentiation, controlling lineage commitment, proliferation, matrix maturation, and mineralization. AMELX, amelogenin; CBFB, core-binding factor beta subunit; CCND1, cyclin D1; CDKN2A, cyclin-dependent kinase inhibitor 2A; COL9A1, collagen type IX alpha 1 chain; FLNB, filamin B; HNRNPLL, heterogeneous nuclear ribonucleoprotein L-like; IGF1, insulin-like growth factor 1; PNO40, partner of NOL1 homolog; RUNX2, Runt-related transcription factor 2; SRSF1, serine/arginine-rich splicing factor 1; TAF4, TATA-box binding protein associated factor 4; VWC2L, von Willebrand factor C domain containing 2 like; WNK1, WNK lysine-deficient protein kinase 1.

During the osteogenic differentiation of BMSCs, the process can be divided into 4 stages: lineage commitment of osteoprogenitor cells, proliferation of pre-osteoblasts, matrix maturation, and matrix mineralization [74,75]. In the lineage commitment stage, filamin B acts as a key upstream RBP, directly influencing the normal development of osteoprogenitor cells by regulating the AS of osteogenesis-related genes *Tpx2* and *Evc* [62]. At the same time, high expression of the TAF4v1 variant together with lnc-PPP2R1B/HNRNPLL synergistically enhances RUNX2 signaling, thereby promoting osteogenic differentiation [64,66]. The 2 variants of Cbfb (Cbfb1 and Cbfb2) serve as co-transcription factors of RUNX2 and exert complementary roles in regulating the transcription of downstream osteogenesis-related genes. Among them, Cbfb2 is indispensable for skeletal development, whereas Cbfb1 is more efficient in enhancing RUNX2 activity [67]. In the proliferation stage, SRSF1 mediates the AS of IGF1 to generate the MGF variant, which activates the ERK signaling pathway to promote pre-osteoblast proliferation [72]. Meanwhile, SRSF1 also regulates the AS pattern of Cyclin D1, thereby maintaining the dynamic balance of cells under a highly proliferative state [73]. During the matrix maturation stage, the up-regulation of the TID-L and TID-I variants enhances the osteoblastic phenotype, characterized by increased alkaline phosphatase activity and calcium deposition [61]. At the same time, the pNO40 variant, which lacks exon 4, promotes the transition of osteoblasts from a “differentiation-proliferative state” to a “matrix maturation state” by down-regulating RUNX2 signaling [63]. In vivo, novel AS patterns of genes such as Col9a1, Wnk1, and CdkN2a can also be observed in mice, suggesting potential involvement in osteogenic maturation, although their precise functions remain to be elucidated [68]. In the matrix mineralization stage, splicing of the amelogenin (AMELX) gene produces miR-exon4, which maintains RUNX2 at an appropriate level to support the sustained progression of matrix mineralization [69]. The 3 AS transcript variants of the *VWC2L* gene (Vwc2l-1, Vwc2l-2, and Vwc2l-3) promote matrix mineralization in MC3T3-E1 osteoblasts, accompanied by increased OSX expression, with Vwc2l-1 showing the strongest effect [76,77]. Moreover, they exhibit differential regulation of markers such as Col1a2 and Bsp, suggesting that they may jointly promote osteogenic differentiation and mineralization through activation of transforming growth factor beta (TGF-β) superfamily signaling [77]. In summary, the 4 stages of osteogenic differentiation are characterized by distinct AS features, with various AS factors and events exerting stage-specific regulatory functions during lineage commitment, cell proliferation, matrix maturation, and matrix mineralization (Fig. 4).

Overall, we observed the broad involvement of AS during osteogenic differentiation, spanning multiple dimensions including RUNX2 transcription factor, noncoding RNAs, and mechanical stimulation. Although existing studies have identified and validated many AS variants related to osteogenic differentiation, such as TID, TAF4, and CBFB2, most remain at the single-gene level, limiting the possibility of constructing a complete AS regulatory network. In the future, under the premise of sufficient sequencing depth and robust AS analysis, exploring the changes in AS regulatory networks during the differentiation of BMSCs into osteoblasts will be an important direction. Moreover, if antisense oligonucleotides (ASOs) or CRISPR tools are applied to in vivo models to target specific AS variants, this will provide the most direct functional validation and causal evidence to clarify their specific roles in osteogenic differentiation.

### Osteoclasts

Osteoclasts are multinucleated cells that are primarily responsible for bone resorption and play a crucial role in maintaining bone homeostasis (Fig. 3). Derived from the monocyte–macrophage lineage, osteoclasts undergo differentiation and maturation into functional cells, a process that is tightly regulated by multiple signaling pathways. Accumulating evidence has demonstrated that AS plays a critical role in osteoclast biology. To date, numerous AS events have been identified to impact osteoclast differentiation. One study has revealed that cytoplasmic polyadenylation element binding protein 4 (CPEB4), an RBP, colocalizes with SRSF5 and SRSF6 through liquid–liquid phase separation, stabilizing the normal AS variant inhibitor of DNA binding 2 (ID2)-201 [78]. In the absence of CPEB4, abnormal variants ID2-202 and ID2-203 are up-regulated, impairing the inhibitory effect of ID2 on osteoclast differentiation and subsequently affecting osteoclastogenesis [78]. Furthermore, a study on multiple myeloma has reported that the exosome-derived AS factor SFRS8 regulates the AS of calcyclin binding protein (CACYBP) in the bone marrow microenvironment. This leads to an increase in CACYBP variant 2 (lacking exon 2) and a concomitant decrease in variant 1 (lacking exon 1), ultimately inducing osteoclast differentiation via the Wnt/β-catenin pathway [79]. The RANKL (receptor activator of NF-kappaB ligand)–RANK (receptor activator of NF-kappaB) signaling pathway is essential for osteoclast survival. Interestingly, a truncated AS variant of RANK, termed vRANK, antagonizes the anti-apoptotic effects of RANKL, thereby inhibiting osteoclastogenesis [80]. Moreover, studies have confirmed that zBTB (zinc finger and BTB domain-containing protein) family proteins, including leukemia/lymphoma-related factor (LRF) and osteoclast zinc finger protein (OCZF), jointly regulate the AS of Bcl, which influences osteoclast survival. In the presence of LRF/OCZF, the degradation of RBP Sam68 (Src-associated in mitosis of 68 kDa) is promoted, resulting in an up-regulation of the Bcl extra large (Bcl-xl; anti-apoptotic) AS variant and a loss of the Bcl extra short (Bcl-xs; pro-apoptotic) variant, thereby maintaining osteoclast survival [81].

Additionally, osteoclast activity is modulated by various hormones and matrix molecules, such as calcitonin, parathyroid hormone, galectins, and protein tyrosine phosphatase-osteoclastic (PTP-oc) [82]. Several studies have demonstrated the involvement of AS in these regulatory processes. For instance, 2 newly identified human calcitonin receptor (CTR) variants, H-CTR5 and H-CTR6, are expressed in giant cell tumors, but their functional role remains unclear [83]. Another research group identified a mouse osteoclast-specific AS variant of the CTR, P3-CTR, which is thought to play an essential role in modulating osteoclast activity [84]. Additionally, 3 alternatively spliced products of parathyroid hormone-related protein (PTHrP) were identified: PTHrP-1-139, PTHrP-1-141, and PTHrP-1-173. Among them, PTHrP-1-139 exhibited the most pronounced osteoclastogenic effects. In a mouse model of breast cancer bone metastasis, mice carrying PTHrP-1-139 exhibit extensive osteolytic lesions and high serum calcium levels [85]. In addition to generating distinct variants via AS, the intronic promoters within glomerular epithelial protein 1 in osteoclasts can drive tissue-specific transcription programs, producing a truncated variant, PTP-oc. This variant regulates tyrosine kinase signaling through dephosphorylation, inhibits RANKL signaling, and contributes to osteoclast survival [86]. The galectin family members (LGALS) are closely related to angiogenesis and consist of 11 members, all of which are expressed at the protein level in humans. LGALS1 inhibits osteoclast activity [87,88]. However, recent studies have identified 2 AS-derived galectin-8 variants in osteoclasts: a long variant (LGALS8-L) and a short variant (LGALS8-S). LGALS8-L, owing to its slower binding kinetics to bone matrix proteoglycans and an additional flexible loop, is proposed to obstruct certain protein interactions, thereby suppressing bone resorption. By contrast, LGALS8-S interacts with more proteins, particularly those associated with secretory vesicles (e.g., lysosome-associated membrane protein 1/2 and chloride channel 7), which are critical for the integrity of the sealing zone and bone resorption activity [89]. AS functions as a switch that determines the ratio of LGALS8-L to LGALS8-S, and directly influences the dynamic balance between osteoclast resorption and migration. Furthermore, a medium-length variant of LGALS8, LGALS8-M, has been identified in endothelial cells. These 3 galectin-8 variants mediate vascular and lymphatic vessel formation by interacting with membrane proteins, such as cluster of differentiation 166 (CD166), integrins, and CD44, suggesting that the AS of LGALS8 in osteoclasts has diverse biological functions [90].

Overall, we found that AS is involved in osteoclast differentiation, survival, and functional regulation, ranging from signaling pathway activity such as RANKL and Wnt/β-catenin, to specific RBP regulation such as CPEB4, SFRS8, and CTR, as well as the shaping of the osteoclast microenvironment such as LGALS8. Mechanistically, AS variants including vRANK, Bcl-xl, and LGALS8-L have been confirmed to exert functional counter-regulatory effects, inhibiting osteoclast activity and acting as “functional switches” for maintaining bone homeostasis. In vitro, through high-throughput sequencing, we propose to further investigate the AS network dynamics during osteoclast maturation, especially under different external stimuli such as mechanical loading and drug intervention. In vivo, by leveraging single-cell sequencing and spatial transcriptomics, monitoring the spatiotemporal RBP changes underlying the formation and maintenance of the osteoclast sealing zone will help to reveal the core functions of AS in bone resorption.

### Chondrocytes

Chondrocytes are the only cell type present in the cartilage tissue and are primarily responsible for maintaining the structural integrity and functional stability of the cartilage [91]. MSC-derived chondrocytes undergo proliferation, differentiation, and maturation during cartilage development, ultimately reaching a terminally differentiated state embedded within the extracellular matrix (ECM) (Fig. 3). This process is strictly regulated by various molecular pathways and characterized by extensive AS events [92]. For instance, one study has demonstrated that TAF4 generates multiple AS variants, including TAF4-v2, TAF4-v4, and TAF4-v5, by altering exons 6 and 7 of the hTAF4-TAFH domain [64]. These variants collectively promote mesenchymal stem cell proliferation and preferentially direct differentiation toward the chondrogenic lineage [64]. Similarly, the AS variant of Wnt-induced secreted protein 1 (WISP1), WISP1v (lacking exon 3), is up-regulated during the terminal differentiation of chondrocytes, suggesting its critical role in the transition to endochondral ossification [93]. Another splice variant, WISP1vx, lacking exons 3 and 4, is thought to be involved in the oncogenic transformation of chondrocytes [93]. Recent studies have revealed that activation of A2AR regulates AS of p53 in chondrocytes, leading to the degradation of full-length p53 and increasing the expression of the anti-aging variant Δ133p53α [94]. Δ133p53α is a p53 AS variant lacking the N-terminal transactivation domain, which resists cellular senescence by antagonizing full-length p53 [94–96]. Δ133p53α mitigates p53-related senescence signaling by down-regulating p21 and p16 or altering their subcellular localization, thereby improving chondrocyte function, delaying cartilage degeneration, and maintaining chondrocyte homeostasis [94]. Similarly, insulin-like growth factor 1 (IGF1) precursors undergo AS to produce the proIGF-1A, proIGF-1B, and proIGF-1C variants [97]. The EA peptide of proIGF-1A (encoded by exons 4 and 5) enhances heparin binding via N-glycosylation, prolonging its extracellular activity and promoting the secretion of IGF-1, which stimulates the synthesis of matrix proteins, such as proteoglycans and type II collagen, facilitating cartilage growth and repair [97]. By contrast, proIGF-1B inhibits IGF-1 secretion without directly enhancing cartilage repair, whereas proIGF-1C, also known as MGF, aids cartilage regeneration by promoting stem cell migration, proliferation, and differentiation through IGF-1 receptor binding [98]. Interestingly, transforming growth factor beta 1 (TGF-β1) signaling mediates spatially distinct AS of superficial zone protein (SZP) in chondrocytes from load-bearing regions of the medial femoral condyle. SZP produces 3 variants: SZP-A (including exons 4 and 5), SZP-B (including exon 4 but lacking exon 5), and SZP-C (lacking exons 4 and 5). The SZP-A and SZP-B variants enhance ECM binding, whereas SZP-C exhibits weaker matrix binding and exists freely in the synovial fluid [99]. AS also plays a critical role in the pathophysiology of the cartilage. A recent study has shown that prednisolone induces AS in glycosyltransferase-like domain-containing protein 1 (Gtdc1) exon 4, producing circ-Gtdc1. Circ-Gtdc1 ubiquitinates and degrades the AS factor SRSF1, thereby inhibiting chondrocyte proliferation and ECM synthesis, ultimately leading to cartilage dysplasia [100]. Supplementation with exogenous circ-Gtdc1 improves joint cartilage quality, providing a potential therapeutic target for early intervention in fetal-origin osteoarthritis (OA) [100].

Chondrocytes and ECM maintain a “symbiotic relationship”, jointly preserving cartilage structure and function. ECM factors markedly influence chondrocytes. Collagen type II alpha 1 chain (COL2A1), a crucial ECM component of cartilage, has 4 AS variants: IIA (COL2A1IIA), IIB (COL2A1IIB), IIC (COL2A1IIC), and IID (COL2A1IID) [101,102]. The abundance of these splice variants changed during chondrocyte differentiation, suggesting a regulatory role at different stages of differentiation [103]. A study demonstrated that disruption of the 5′ SS in COL2A1 exon 2 suppresses IIC expression, increases IIA and IID mRNA levels, and decreases IIB mRNA levels, resulting in enhanced expression of embryonic type II collagen in the ECM. This indicates a functional regulatory role for COL2A1 exon 2 [104]. Another essential ECM component, fibronectin 1 (FN1), has garnered attention due to its role in chondrocyte maturation via AS regulation. Specifically, TGF-β1 modulates the AS of SRp40 (SRSF5), generating 2 variants, SRp40 and SRp40LF. During the proliferative phase, SRp40 promotes FN1 variants containing extra domain A (FN1-EDA), thereby accelerating chondrocyte maturation [105]. Conversely, SRp40LF inhibits the FN1-EDA variant, suggesting that the balance between these variants is a critical AS regulatory node for chondrocyte maturation [105]. Recent studies have shown that the RNA helicase DDX5 directly participates in the AS process by regulating FN1 AS to suppress the FN1-EDA variant, thereby reducing cartilage fibrosis [106]. DDX5 also regulates procollagen-lysine, 2-oxoglutarate 5-dioxygenase 2 (PLOD2) AS by inhibiting the production of the full-length PLOD2 variant (PLOD2-WT) while maintaining PLOD2-SE14 expression, reducing fibrosis-associated collagen crosslinking and preventing cartilage fibrosis [106]. Furthermore, another FN1 AS variant, FN1-208, was differentially expressed between osteoarthritic and normal cartilage samples. Further studies have revealed that the up-regulation of FN1-208 negatively affects ECM deposition in chondrocytes, warranting further investigation into its functional role [107].

Overall, we observed that specific AS events in cartilage components, such as WISP1, IGF1, COL2A1, and FN1, are associated with chondrocyte development and functional phenotypes. The SRSF5-derived variants SRp40 and SRp40LF display temporal expression and functional antagonism, respectively dominating the proliferative and maturation phases of chondrocytes, and serve as a key AS checkpoint regulating cartilage development. However, most studies remain at the single-gene level and lack a systematic positioning of AS events within regulatory networks. The interactions of AS with epigenetic modifications, post-translational modifications, and noncoding RNAs in chondrocytes have not yet been systematically explored. Moreover, under pathological conditions such as OA or rheumatoid arthritis, systematically dissecting AS patterns in chondrocytes and validating functional targets will represent a promising research direction.

### Bone matrix

The bone matrix comprises various components, including matrix proteins, collagen fibers, noncollagenous proteins, and proteoglycans [108,109]. The physical properties of matrix proteins are altered by AS (Fig. 3), proteolytic processing, and the formation of homo- and hetero-oligomers [110]. Covalently crosslinked type II collagen is the major collagen component of the cartilage ECM, providing tensile strength to the cartilage. The protein encoded by COL2A1 forms homotrimeric procollagen, which undergoes proteolytic processing to assemble crosslinked fibers in the ECM of cartilage tissue [111]. Exon 2 of COL2A1 encodes a conserved domain of 69 amino acids in the NH_2_-terminal region of type IIA procollagen [112]. Cartilage progenitor cells predominantly produce variants containing exon 2 (types IIA and IID), whereas differentiated chondrocytes mainly generate variants lacking exon 2 (type IIB) [104,112]. Types IIA and IID, which are embryonic variants, are coexpressed during chondrogenesis and dominate the early stages of embryonic development before being down-regulated [113]. Type IIB is the only variant expressed and synthesized by fully differentiated chondrocytes and is more abundant in the epiphyseal cartilage of late embryonic and postnatal mice [113]. Homozygous mice engineered to retain exon 2 of type IIA showed persistent expression of the type IIA protein in the trabecular bone in the subepiphyseal and metaphyseal regions, without apparent effects on embryonic development [114]. Proliferative and hypertrophic chondrocytes, which typically synthesize type IIB procollagen, develop normally even when producing only type IIA procollagen [114].

Exon 2 of COL2A1 also contains an additional A5SS, resulting in a truncated mRNA variant (type IIC). In vivo suppression of type IIC AS using site-directed mutagenesis revealed no skeletal or gross morphological differences but led to elevated levels of variant type IIA and IID procollagen and reduced levels of type IIB variants in differentiated chondrocytes, indicating that type IIC AS is indispensable for ECM maturation in cartilage [104]. Antibodies targeting peptide sequences spanning exons 1 to 3 of mouse COL2A1, such as IIBN, which specifically recognizes type IIB proteins, have been developed. Using IIBN, high type IIB procollagen levels were detected in the hypertrophic chondrocytes of wild-type neonatal mice, whereas no signals were observed in the resting or proliferative zones, suggesting that chondrocytes efficiently transported type IIB procollagen from the endoplasmic reticulum to the bone matrix [115]. Another antibody specific to type IIB, pNIIB52, is effective for detecting type IIB in human, canine, and bovine cartilage, but fails to detect type IIB in mouse or rat cartilage owing to differences in the exon 1/3 sequences targeted by the antibody [113]. Owing to limitations in conventional AS detection methods, where the amplification efficiencies of polymerase chain reaction (PCR) products of different sizes tend to vary, researchers have developed alternative transcript-qPCR (AT-qPCR) based on a standard plasmid containing multiple amplicons. Using AT-qPCR, we observed that insulin-induced chondrogenic differentiation in ATDC5 cells primarily expressed types IIA and IID [116]. Compared to conventional PCR, AT-qPCR yields a lower IIB/IIA product ratio, which is closer to the true results [116]. Improving AS detection methods represents a key direction for future research.

The matrilin family, a critical component of the cartilage ECM, mediates interactions between collagen fibers and other matrix components [117]. An AS event between the second vWFA domain and the coiled-coil domain of matrilin-2 (encoded by matrilin-2 [MATN2]) gives rise to 2 variants: a short variant (MATN2S) and a long variant (MATN2L) [118]. The long variant of matrilin-2 can form multimers, whereas the short variant can only form oligomers, thereby affecting its post-translational modifications [118]. Alterations in the matrilin family are involved in abnormalities in the molecular network of the bone matrix.

Overall, the different AS variants of type II collagen represented by COL2A1 (IIA, IIB, IIC, and IID) are not expressed randomly, but rather undergo highly programmed spatiotemporal transitions, which constitute one of the reasons ensuring proper development and maturation of the bone matrix. The study of AS function should not be limited to its regulation of gene expression levels, but should also focus on a new dimension—how AS reshapes protein domain composition to regulate protein conformation, oligomeric state, and functional activity. MATN2, which generates variants with distinct polymerization capacities through AS, exemplifies this regulatory layer. By using cryo-electron microscopy to observe structural differences among AS variants at the protein conformation level, a critical frontier direction will emerge. Traditional PCR methods are limited by amplification efficiency bias, making it difficult to accurately quantify the proportion of AS variants, while the emerging AT-qPCR technique, though improved, still cannot achieve in situ detection of AS variants at single-cell resolution. As a result, we are not yet able to precisely define the distribution and functions of AS variants across the distinct zones of cartilage differentiation—resting, proliferative, hypertrophic, calcified, and ossification—or directly connect AS events with specific cellular states. The development of new AS detection technologies with single-cell and spatial resolution will be essential to advance mechanistic insights in this field.

### Bone remodeling

Although we have discussed in detail the AS features of osteogenic cells and the bone matrix, the role of AS in dynamic bone remodeling has not yet received sufficient attention. Bone remodeling in humans is considered a cell optimization process essential for survival and is influenced by multiple factors, including osteocytes, mechanical stress, the RANKL/osteoprotegerin (OPG) ratio, PTH, estrogen, TGF-β1, and vitamin D [119,120]. RANKL, encoded by *TNFSF11*, is primarily secreted by osteoblasts, bone marrow stromal cells, T cells, and synovial cells. It binds to RANK on the surface of osteoclast precursors and mature osteoclasts, activating downstream signals such as NF-κB and thereby promoting osteoclast differentiation, maturation, and bone resorption [121]. As a regulatory mechanism, osteoblasts under physiological conditions—including bone homeostasis, mechanical stimulation, or estrogen signaling—express OPG (encoded by *TNFRSF11B*), a high-affinity soluble decoy receptor that binds RANKL, preventing its interaction with RANK and inhibiting osteoclast activation to maintain remodeling balance [122]. In addition to osteoblasts, OPG is also secreted by various other tissues, including the heart, liver, and B cells. Its expression is regulated by hormones, inflammatory cytokines, and signaling pathways such as Wnt/β-catenin and Notch. OPG is often insufficiently up-regulated in response to increased RANKL expression, resulting in an elevated RANKL/OPG ratio and enhanced osteoclastogenic activity [123]. Taken together, the RANKL–RANK–OPG axis serves as the core regulatory system of bone remodeling, integrating endocrine, immune, and mechanical signals through precise modulation of osteoclast activity to maintain bone homeostasis and structural renewal.

RANKL exists in 2 forms: membrane-bound and soluble (sRANKL). Systematic analyses have shown that sRANKL is mainly generated by extracellular proteolytic cleavage of membrane-bound RANKL via metalloproteinases such as MMP-7 or a disintegrin and metalloprotease 17 (ADAM17), representing a post-translational regulatory mechanism [124]. In bone tissue, the Tnfsf11 gene undergoes AS to produce 3 major transcript variants: RANKL1 (full-length), RANKL2 (partial deletion of exon 2, resulting in a truncated intracellular domain), and RANKL3 (lacking exons 2 to 4, encoding a soluble RANKL and constituting one of the sources of sRANKL) [125,126]. Classical evidence comes from multiple myeloma cell lines, which secrete active sRANKL through AS and post-translational modifications, thereby inducing TRAP^+^ multinucleated cell formation from human or mouse bone marrow mononuclear cells and promoting osteoclastogenesis [127]. Unlike interleukins, PTH under sustained stimulation uniquely induces Tnfsf11 expression while simultaneously facilitating proteolytic processing of full-length RANKL to release sRANKL into the extracellular space [124]. The AS variant ΔCAPRI, which lacks one exon within the RasGAP domain, is highly expressed in primary mouse osteoblasts but exhibits low expression in osteoclasts [128]. ΔCAPRI activates the Ras/matrix metallopeptidase 14 (MMP14) axis and enhances RANKL shedding, thereby increasing sRANKL production and ultimately influencing bone remodeling [128]. Collectively, RANKL AS variants cooperatively regulate sRANKL production, bridging transcriptional and post-translational mechanisms as a key regulatory module in bone remodeling.

Three AS variants of RANK have been identified: RANK-a (lacking exon 9), RANK-b (lacking exons 8 and 9), and RANK-c (lacking exons 7 to 9) [129]. RANK-c lacks NF-κB activation capability and, when co-expressed with full-length RANK, suppresses downstream signal transduction, suggesting that it serves as an intrinsic negative regulator of the RANK pathway. However, the specific AS factors involved in generating these variants remain unknown [129]. In murine RAW264.7 cells, a truncated RANK splice variant named vRANK has been identified. This variant contains a novel exon 2a inserted between exons 1 and 2, which introduces a premature stop codon, resulting in a protein lacking the transmembrane and intracellular domains and retaining only part of the signal peptide [129]. Compared to full-length RANK, vRANK fails to activate the NFATc1 signaling pathway, strongly inhibits osteoclast formation, and promotes apoptosis, possibly by competing with RANK for RANKL binding and functioning as an endogenous antagonist of RANKL signaling [129]. In human brain tissue, a 42-bp splice variant named RANK-e5a lacking exon 5 has been identified. This variant alters the conformation of the TNFR cysteine-rich domain, reducing its RANKL binding affinity [130]. Although RANK-e5a retains the intracellular domain, its shortened extracellular domain weakens the ability to activate downstream NF-κB signaling upon RANKL binding [130].

In addition, Periostin-4, an AS variant of Periostin lacking exons 17 and 21, has been identified as the predominantly expressed isoform in primary human osteoblasts. Functionally, Periostin-4 acts as an extracellular signal integrator that substantially promotes osteoblast differentiation and mineralization in vivo [131]. To date, only a single transcript has been identified for OPG, and no AS variants have been reported.

**Fig. 5.**
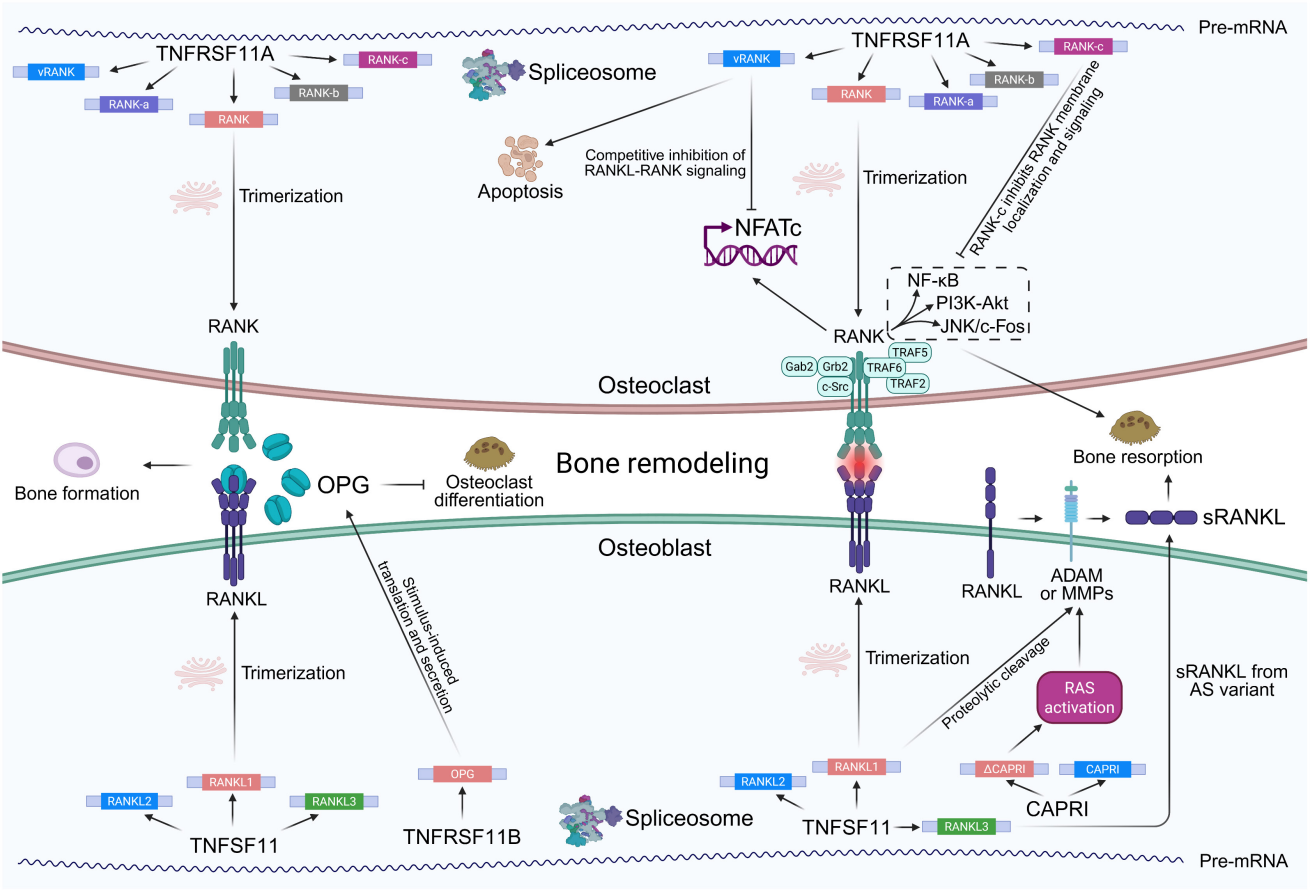
Alternative splicing regulates the RANKL–RANK–OPG axis during bone remodeling. The upper part shows splicing-derived RANK variants (e.g., vRANK, RANK-a, RANK-b, and RANK-c), which modulate receptor trimerization, membrane localization, and downstream signaling pathways such as NF-κB, PI3K-Akt, and JNK/c-Fos. The lower part illustrates AS events generating different RANKL isoforms (e.g., RANKL1, RANKL2, RANKL3, and ΔCAPRI) which affect secretion, proteolytic cleavage, and the production of soluble RANKL (sRANKL). The central panel highlights how these AS-derived variants interact with RANK and OPG in osteoclasts and osteoblasts to regulate osteoclast differentiation, bone resorption, and bone formation. ADAM, a disintegrin and metalloproteinase; Akt, protein kinase B; CAPRI, calcium-promoted Ras inactivator; JNK, c-Jun N-terminal kinase; MMPs, matrix metalloproteinases; NF-κB, nuclear factor kappa-light-chain-enhancer of activated B cells; NFATc, nuclear factor of activated T cells, cytoplasmic component; OPG, osteoprotegerin; PI3K, phosphoinositide 3-kinase; RANK, receptor activator of nuclear factor kappa-B; RANKL, receptor activator of nuclear factor kappa-B ligand; sRANKL, soluble receptor activator of nuclear factor kappa-B ligand; TNFRSF11A, tumor necrosis factor receptor superfamily member 11A; TNFRSF11B, tumor necrosis factor receptor superfamily member 11B; TNFSF11, tumor necrosis factor superfamily member 11.

Overall, we observed that the classical RANKL–RANK–OPG signaling axis of bone remodeling is also regulated by AS, highlighting that AS not only functions at the level of post-transcriptional modification but also plays an important role in intercellular communication (Fig. 5). Mechanistically, the upstream RBP-mediated AS regulation of the RANKL–RANK–OPG axis and its temporal dynamics remain insufficiently explored and hold important research value. In the future, approaches such as cross-linking and immunoprecipitation sequencing (CLIP)-seq and CRISPR screening will help to construct a more comprehensive RBP network of bone remodeling. For target validation, an important direction will be to generate gene-edited mouse models of AS variants, for example, by specifically deleting vRANK, to observe the net effects on bone remodeling homeostasis and bone loss under pathological conditions. Such animal models will not only reveal the independent functions of AS variants but also provide new experimental tools for studying bone metabolic diseases such as osteoporosis, thereby laying the foundation for therapies targeting AS variants or small molecules.

### Distinct AS in bone and the musculoskeletal system

Compared with other tissues, the characteristics of tissue-specific AS in the musculoskeletal system remain an intriguing area of study. Brinegar et al. [132] conducted RNA sequencing (RNA-seq) across different developmental stages of mouse skeletal muscle—including E18.5 (embryonic day 18.5), PN2 (postnatal day 2), PN14, PN28, and adulthood (22 weeks)—to investigate dynamic changes in AS. Their results showed that AS events were most dynamic between PN2 and PN14. Among the 768 AS events identified between E18.5 and adulthood, 32% occurred within the first 2 postnatal weeks, with ES accounting for 77% of them. These events were significantly enriched in calcium-handling genes [132]. Notably, 3 AS variants of calcineurin A genes exhibited a transition from fetal-type to adult-type splicing patterns after birth. This transition involved increased inclusion of exons 10a and 13, which altered the conformation of the autoinhibitory domain and calmodulin-binding region, thereby modulating phosphatase activity and calcium sensitivity [132,133]. Collectively, these findings suggest that AS of calcium signaling-related genes constitutes a key component of tissue-specific AS regulatory networks, although more direct experimental evidence is needed.

Bone cells are responsible for regulating more than 99% of the body’s calcium content. Calcium functions as a hormonal and cytokine-like regulator that participates in intercellular communication and cell–matrix interactions [134]. At least 6 classes of calcium channels have been identified in osteoblasts and osteoclasts, including voltage-gated calcium channels (VGCCs), store-operated calcium entry channels, transient receptor potential channels, ATP-gated P2X channels, inositol 1,4,5-triphosphate receptors, and mechanically activated Piezo channels. These channels involve at least 18 genes that cooperatively regulate intracellular calcium homeostasis [135]. Among them, *CACNA1C*, a member of the VGCC family, is highly expressed in both chondrocytes and osteoblasts. Conditional knockout of *CACNA1C* in mice leads to abnormal endochondral ossification, characterized by shortened limbs and digital defects [136,137]. Exons 8 and 8a of *CACNA1C* are mutually exclusive, and the G406R mutation in exon 8a enhances OPG secretion, thereby suppressing osteoclast activity and altering skeletal phenotypes [136,138]. These findings indicate that AS of *CACNA1C* plays a critical role in the fine-tuning of VGCC function in bone-specific contexts. Similarly, *P2RX5*, a member of the P2X receptor family, is highly expressed in osteoclasts and plays a role in bone immune regulation [139]. The AS site at exon 10 of the human *P2RX5* gene is influenced by a single-nucleotide polymorphism, where the G allele (as opposed to the T allele) promotes ES, resulting in an AS variant lacking key structural domains [139,140]. The resulting truncated *P2RX5* variant encodes a protein that fails to localize to the plasma membrane and cannot form a functional ATP-gated calcium channel, thus losing its capacity to mediate calcium influx [141,142]

Piezo2, a mechanosensitive ion channel, mediates mechanical sensing in bone tissue and plays a role in skeletal development [143]. To date, at least 17 AS variants of Piezo2 have been identified, primarily involving exons 10, 18, 19, 33, 35, and 40 [144]. In neurons, Piezo2 variants containing exon 35 (E35) exhibit faster inactivation kinetics and greater mechanical responsiveness [144]. Notably, E35 is highly expressed in bone-associated sensory neurons (e.g., Ntrk3^+^ cells), but nearly absent in nociceptors (e.g., Mrgprd^+^ cells), suggesting that bone afferents are proposed to regulate Piezo2 responsiveness and mechanosensitivity through AS [144]. In addition, a novel AS variant of Piezo1, termed Piezo1.1, has been identified in mouse myogenic precursor C2C12 cells. This variant results from skipping of exon 30 and exhibits enhanced mechanical sensitivity and ion conductivity [145]. The tissue-specific splicing of calcium channel genes in bone is primarily governed by RBPs with bone cell-restricted expression, while mechanical stimulation likely acts as a critical modulatory factor. Such adaptive AS may also occur, to some extent, in other cells responding to mechanical stimulation or calcium fluxes, such as cardiomyocytes and vascular smooth muscle cells. However, the occurrence and functional relevance of Piezo1/2 AS events in osteoblasts and osteoclasts remain poorly studied, and direct evidence is still lacking.

Taken together, the AS characteristics of calcium channel genes serve as potential molecular signatures of tissue-specific AS regulation. Nevertheless, the detection of AS events largely relies on high-throughput RNA-seq, which demands sufficient sequencing depth to ensure sensitivity and accuracy [146]. Under physiological conditions, bone tissue is densely mineralized, rich in hydroxyapatite and other inorganic components, and characterized by low cellularity. These properties limit the efficiency of high-quality RNA extraction in vivo, thereby posing challenges for AS profiling and downstream transcriptomic analyses [147]. With the growing accumulation of bone tissue-related single-cell datasets, systematically resolving the AS patterns of distinct bone cell subpopulations will facilitate deeper secondary analyses of these data and yield novel mechanistic insights [148–154]. The characteristic AS patterns of bone cell subpopulations will represent an important direction for future investigation.

## AS Impact on Bone Tumors

### Osteosarcoma

**Fig. 6.**
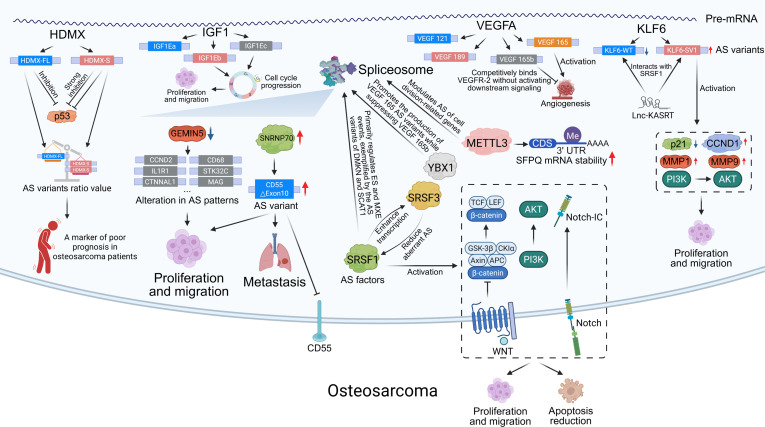
Aberrant alternative splicing and RBP regulatory networks in osteosarcoma. On the left, AS variants of HDMX and IGF1 drive cell proliferation and migration, with HDMX variant ratios serving as prognostic markers. In the center, splicing factors (e.g., YBX1, SRSF3, and SRSF1) and RNA modifications (e.g., METTL3-mediated regulation of SFPQ mRNA stability) modulate widespread AS patterns, influencing tumor growth and signaling. On the right, VEGFA and KLF6 variants promote angiogenesis and activate PI3K-AKT signaling to enhance invasion. At the bottom, CD55 ΔExon10 and pathway-level alterations such as WNT/Notch signaling contribute to metastasis, proliferation, and apoptosis resistance. AKT, protein kinase B; CCND1, cyclin D1; CD55, complement decay-accelerating factor; GEMIN5, gem-associated protein 5; HDMX, MDM4 p53 regulator; IGF1, insulin-like growth factor 1; IGF1EB, insulin-like growth factor 1 enhancer binding protein; KLF6, Kruppel-like factor 6; METTL3, methyltransferase like 3; MMP1, matrix metalloproteinase 1; PI3K, phosphoinositide 3-kinase; SNRNP70, small nuclear ribonucleoprotein U1 subunit 70; SRSF1, serine/arginine-rich splicing factor 1; SRSF3, serine/arginine-rich splicing factor 3; VEGFA, vascular endothelial growth factor A; YBX1, Y-box binding protein 1; p21, cyclin-dependent kinase inhibitor 1.

Beyond essential roles in maintaining bone homeostasis under physiological conditions, AS also contributes notably to the pathogenesis of bone tumors. OS is the most common primary malignant bone tumor and is highly prone to distant metastasis [155]. AS and RBPs are involved in various OS progression processes (Fig. 6). RNA-seq results from 18 OS and adjacent nontumor samples revealed differences in leptin receptor overlapping transcript (LEPROT) gene variants between tumor and adjacent nontumor tissues [156]. Five LEPROT variants are overexpressed in OS tissues, whereas 22 variants are overexpressed in adjacent tissues, indicating that LEPROT is relatively down-regulated in OS tissues [156]. This highlights the importance of comprehensively considering all the variants generated by AS and their overall trends when evaluating whether a gene is up-regulated or down-regulated during OS. Overexpression of U1 small nuclear ribonucleoprotein 70K (SNRNP70), a component of the spliceosome, promotes OS migration, whereas its knockdown inhibits metastasis in vivo [157]. SNRNP70 facilitates exon 10 skipping in CD55, producing a shorter variant that enhances OS cell proliferation, migration, and metastasis [157]. In OS cell lines with low metastatic potential, AS-related proteins such as apoptosis chromatin condensation inducer 1, poly(A)-binding protein, heterogeneous nuclear ribonucleoprotein A2/B1, block of proliferation 1, and gem-associated protein 5 (GEMIN5) are up-regulated [158]. GEMIN5, a critical component of the spliceosome snRNP, affects OS cell migration when either overexpressed or knocked down [158].

SRSF1, a trans-acting factor that regulates AS, also influences tumor behavior in OS. Markedly up-regulation of SRSF1 expression was observed in OS samples and cell lines. SRSF1 overexpression enhances migration and invasion, while reducing apoptosis, whereas SRSF1 knockdown produces the opposite effects [159]. SRSF1 primarily triggers SE and MXE events, and is closely associated with the Wnt, NOTCH, and PI3K (phosphoinositide 3-kinase)–protein kinase B (AKT) pathways. SRSF1 knockdown altered the variant ratios of dermokine and SCAT1 (small cysteine-rich protein 1) [159,160]. Interestingly, the AS factors SRSF3 and SRSF1 were coexpressed in OS with mutual regulatory interactions. They synergistically regulate the AS of numerous genes associated with cell proliferation and cell cycle [161]. SRSF3 prevents cryptic intron missplicing in SRSF1 exon 4, ensuring the expression of normal SRSF1 variants, while SRSF1 promotes SRSF3 transcription without altering its AS pattern [161]. This mutual regulation between AS factors plays an important role in OS progression.

Vascular endothelial growth factor A (VEGFA)-AS events provide another representative example of OS. AS of VEGFA exons 5 to 8 generates variants with different amino acid lengths, such as VEGF 121, VEGF 165, and VEGF 189 [162]. AS at the 3′ SS of the terminal exon in VEGF 165 produces the VEGF 165b variant, which binds to VEGFR-2 but does not activate signaling pathways, thereby indirectly inhibiting the pro-angiogenic function of VEGF 165 [163,164]. In OS, the splicing factor Y-box binding protein 1 (YBX1) promotes the production of VEGF 165 variants while suppressing VEGF 165b, ultimately driving OS progression [165]. YBX1 also regulates the AS of DDX3X and contributes to the progression of OS [166].

Methyltransferase-like 3 (METTL3), METTL14, and Wilms tumor 1-associated protein (WTAP), key m6A modifiers, also regulate AS in OS [167]. In U2OS cells, knockdown of METTL3 induces widespread AS alterations involving 1,803 genes, which are enriched in cell cycle-related pathways, suggesting that METTL3 may play a critical role in cell cycle control of bone tumors through AS regulation [168]. METTL3 also promotes m6A modification of the 3′ UTR of the splicing factor proline- and glutamine-rich (SFPQ) mRNA, enhancing its stability [168]. In addition, previous studies have systematically elucidated the functional interplay between m6A modification and AS in cancer [169]. Methylated RNA immunoprecipitation sequencing data revealed that over 70% of AS sites within 200 bp were associated with m6A peaks mediated by METTL14, which enhances OS tumorigenicity by promoting m6A modifications in the coding region of meningioma 1 (MN1) [167,170]. Interestingly, AS in OS also produces noncoding RNAs that influence tumor progression. An intronic region of SRSF1 generates a 280-bp Lnc-RNA (termed Lnc-KASRT) via AS, which promotes SRSF1 binding to Krüppel-like factor 6 (KLF6) pre-mRNA, resulting in more KLF6 splice variant 1 (KLF6-SV1) than the wild-type KLF6 variant [171]. KLF6-SV1 enhances OS cell migration, invasion, and tumorigenic capacity by down-regulating p21 and up-regulating CCND1, MMP1, and MMP9 expression [171]. KLF6-SV1 also affects the PI3K/AKT pathway [172].

IGF1, widely studied in OS, also undergoes extensive AS with all its variants containing exons 3 and 4, whereas AS occurs primarily in exons 5 and 6 [173]. IGF1 consists of 6 exons and 5 introns, encoding IGF1Ea (including only exon 6), IGF1Eb (including only exon 5), and IGF1Ec variants (including both exons 5 and 6) [173,174]. Different OS cell lines exhibit varying dominant IGF1 variants. For instance, MG63 expresses only IGF1Ea and IGF1Ec, with IGF1Ec expression being higher than that in MNNG-HOS and HOS. IGF1Ec promotes cell cycle progression in MG63 cells, enhancing their proliferation and migration. U2OS cells predominantly express IGF1Eb. Both IGF1Eb and IGF1Ec enhance OS malignancy, whereas IGF1Ea did not. Overall, the proportion and functions of IGF1 AS variants vary among OS cell lines and warrant further investigation. Interestingly, dihydrotestosterone enhances IGF1Ea and IGF1Ec expression and induces IGF1Eb expression in MG63 cells, thereby partly explaining the more severe OS in male adolescents [175]. In OS, IGF1 variants differ fundamentally from those in osteoblasts, such as MGF. MGF can be regarded as a specialized form of IGF1Ec, whose transient induction exerts a functional role in skeletal repair. By contrast, the sustained overexpression of IGF1Ec and IGF1Eb in OS drives oncogenic processes. Moreover, mechanical stimulation in osteoblasts is intermittent, enabling a self-limiting reparative response, whereas pathological stimulation in OS is persistent, leading to irreversible activation of tumor-promoting pathways. Accordingly, the AS pattern of IGF1 in osteoblasts reflects an adaptive and transient response, while in OS, it is locked into a constitutive pro-tumorigenic state.

Studies of *TP53* mutations in OS revealed that approximately 20% of the cases exhibit somatic rearrangements with breakpoints in intron 1, primarily upstream of the Hp53int1 variant [176,177]. In 50% of OS cases, intron 1 frameshift alterations were detected, whereas the coding sequence remained unchanged, suggesting that p53 mutations lead to abnormal AS, producing dominant-negative p53 variants that impair the normal function of p53 tetramers [178,179]. AS also plays a critical role in the response to genotoxic stress. Previous studies have indicated that HPV16 E6 overexpression in U2OS cells enhances AS activity. Although it did not alter sensitivity to mitomycin C treatment, it up-regulated the AS factors SFRS6 and SFRS7 [180], suggesting that AS is implicated in regulating the response to external damage or stress in the absence of p53. Human double minute X (Hdmx), an inhibitor of p53, also exhibits abnormal AS patterns in OS. The exclusion of exon 6 generated a truncated protein, Hdmx-S, with a higher p53 binding affinity, accompanied by a reduction in full-length Hdmx (Hdmx-FL) [181,182]. High Hdmx-S/Hdmx-FL ratios are associated with rapid metastatic progression and poor overall survival in patients with high-grade OS, making them more effective prognostic markers than p53 mutations [183]. This is attributable to elevated Hdmx-S levels driven by abnormal AS in p53-inactivated OS, which promotes tumor growth [183,184].

Interleukin-24 (IL-24) has garnered attention for its ability to reduce OS proliferation and stemness via Notch and Wnt/β-Catenin signaling, without affecting normal cells [185,186]. In U2OS, IL-24 undergoes AS to produce 7 variants that primarily affect exons 2, 3, and 5, which are regulated by SFRS6. SFRS6 overexpression increases the proportion of IL-24 variants lacking exons 2, 3, and 5 [187]. These variants activate caspases 3 and 7, inducing apoptosis in U2OS cells, but not in nontumor cell lines, such as NOK [187]. Targeting specific IL-24 AS variants represents a promising therapeutic strategy for the treatment of OS.

### Ewing sarcoma

**Fig. 7.**
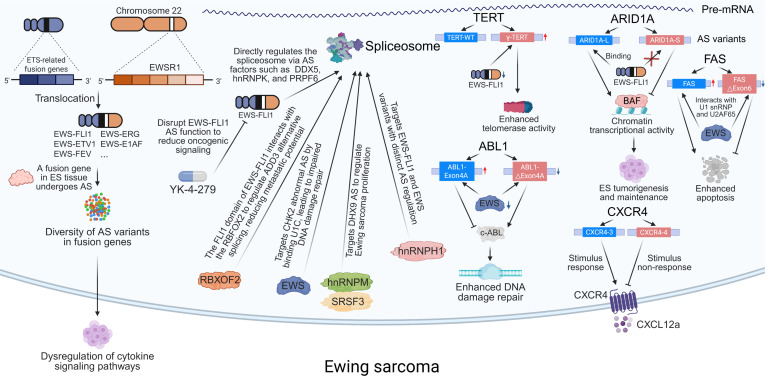
Alternative splicing and fusion gene regulatory networks in Ewing sarcoma. On the left, chromosomal translocations generate ETS-related fusion genes (e.g., EWS-FLI1 and EWS-ERG), which undergo diverse AS events and disrupt cytokine signaling pathways. In the center, the EWS-FLI1 fusion protein directly regulates spliceosome components and AS factors (e.g., DDX5, hnRNPK, and PRPF6), altering splicing programs that drive EwS cell proliferation, DNA damage repair, and survival. On the right, AS variants of downstream targets, such as TERT (enhanced telomerase activity), ABL1 (DNA repair), ARID1A (chromatin regulation), CXCR4 (stimulus response), and FAS (apoptosis), contribute to EwS tumorigenesis, maintenance, and therapeutic resistance. ABL1, ABL Abelson tyrosine-protein kinase 1; ARID1A, AT-rich interaction domain 1A; CXCR4, C-X-C motif chemokine receptor 4; DDX5, DEAD-box helicase 5; EWS, Ewing sarcoma breakpoint region 1 fusion protein; EWSR1, EWS RNA binding protein 1; FAS, Fas cell surface death receptor; hnRNPA1, heterogeneous nuclear ribonucleoprotein A1; hnRNPH1, heterogeneous nuclear ribonucleoprotein H1; hnRNPM, heterogeneous nuclear ribonucleoprotein M; PRPF6, pre-mRNA processing factor 6; RBFOX2, RNA binding Fox-1 homolog 2; SRSF3, serine/arginine-rich splicing factor 3; TERT, telomerase reverse transcriptase.

EwS is a malignant bone tumor that primarily affects adolescents between 10 and 19 years of age [188]. Its malignancy is predominantly driven by FET-ETS (fused in Ewing tumor family–erythroblast transformation-specific) fusion genes, resulting from the fusion of FET family genes with ETS family genes within tumor cells. This fusion leads to oncogenic transformation and malignant progression by disrupting the cellular physiological signaling network [188]. According to current knowledge, AS occurs across multiple processes of malignant progression of EwS, including the oncogenic fusion gene itself (Fig. 7). For instance, one study identified 9 AS variants of EWS-FLI1 (Ewing sarcoma friend leukemia integration 1) and one AS variant of EWS-ERG (Ewing sarcoma erythroblast transformation-specific gene) fusion genes in 23 EwS tissue samples, involving EWSR1 (Ewing sarcoma RNA binding protein 1) exon 8 and multiple exons of FLI1, such as exons 5 and 8 [189]. Approximately 21.7% of EwS tissues harbor multiple fusion variants, often with at least one in-frame variant capable of encoding functional oncogenic proteins [189]. The diverse AS variants of fusion genes in EwS affect growth factor levels and cytokine signaling pathways, thus playing a crucial role in EwS pathogenesis.

Although various oncogenic fusion proteins have been identified in EwS, more than 80% of cases involve the EWS-FLI1 fusion protein, which primarily functions as a transcription factor that dysregulates target gene expression. AS plays an important role in EWS-FLI1-induced oncogenic transformation. Proteomic studies targeting AS in EWS-FLI1 have shown that EWS-FLI1 integrates into the spliceosome network and directly interacts with several AS factors, including DDX5, hnRNPK, and PRPF6, to regulate the AS of target genes, such as CLK1 (CDC-like kinase 1), CASP3 (caspase-3), PPFIBP1 (protein tyrosine phosphatase fibronectin type III domain-containing 1), and TERT (telomerase reverse transcriptase). Compared to wild-type TERT (TERT-WT), AS of TERT induced by EWS-FLI1 down-regulation generates the γ-TERT variant (lacking exon 11), which exhibits higher telomerase activity, potentially reflecting a stress compensation mechanism of EWS-FLI1 [190]. Similarly, EWS-FLI1 directly binds the ARID1A-L variant (including exon 8) rather than the ARID1A-S variant (lacking exon 8) to form a stable chromatin remodeling BRG1/BRM-associated factor complex, supporting EwS cell proliferation and survival [191]. Reduced ARID1A-S expression decreases EWS-FLI1 stability, disrupts chromatin remodeling, and suppresses tumor growth [191,192]. The YK-4-279 inhibitor, which disrupts the interaction between EWS-FLI1 and ARID1A-L, induces ARID1A-S expression, leading to apoptosis, highlighting the critical regulatory role of ARID1A variants in EwS development [191]. The EWS domain of the EWS-FLI1 fusion protein is closely associated with AS. EWS recruits U1snRNP (U1 small nuclear ribonucleoprotein) and U2AF65 to the splice site of exon 6 of FAS (Fas cell surface death receptor), promoting their inclusion and enhancing FAS-mediated apoptosis [193]. By contrast, EWS-FLI1 shows reduced activity at FAS splice sites, suppressing the production of pro-apoptotic variants and aiding EwS cells to evade apoptosis [193]. Ultraviolet (UV) radiation induces the lysine acetylation of EWSR1. This modification inhibits its binding to U1 small nuclear ribonucleoprotein C (U1C), a spliceosomal component, and consequently disrupts the inclusion of exon 6 in checkpoint kinase 2 (CHK2). This leads to aberrant CHK2 splicing and impaired DNA damage repair [194]. The loss of exon 6 reduces CHK2 activity, impairs DNA damage response signaling, and promotes carcinogenesis [195]. Interestingly, UV exposure weakens the binding of EWS to target RNAs and induces transient accumulation in the nucleoli. This process triggers AS changes in ABL proto-oncogene 1 (ABL1), CHK2, and mitogen-activated protein kinase kinase kinase kinase 2 (MAP4K2), which regulate DNA damage responses [196]. Specifically, the absence of EWS alters 5′ SS selection of ABL1 exon 3, increasing the ABL1-Exon4A variant while decreasing the ABL1-ΔExon4A variant. ABL1-Exon4A reduces functional Abelson tyrosine kinase (c-ABL) protein levels, impairing DNA repair capacity, whereas ABL1-ΔExon4A maintains normal c-ABL function, aiding cell survival after DNA damage. Similarly, EWS maintains the normal inclusion of MAP4K2 exon 9, preserving MAP4K2 kinase activity and its role in stress response signaling. Overall, EWS plays a pivotal role in UV-induced genotoxic stress by specifically regulating the AS of ABL1, CHK2, and MAP4K2, thus affecting DNA damage responses and cell fate decisions. The formation of EWS-FLI1 in EwS attenuates EWS-mediated protection of DNA repair via AS regulation [197].

More recently, a study revealed that the FLI1 domain of EWS-FLI1 also mediates AS of target gene pre-mRNAs. It interacts with the AS factor RBFOX2 through the conserved C-terminal domain of FLI1 to regulate the AS of adducin 3, increasing the expression of its long variant and inhibiting the mesenchymal phenotype of ES cells, thereby reducing the metastatic potential [198]. The anti-EWS-FLI1 drug YK-4-279 disrupts the interaction between EWS-FLI1 and protein chaperones p68 (encoded by *DDX5*) and RHA, impairing EWS-FLI1-induced AS and downstream oncogenic signaling [190]. Similarly, YK-4-279 promotes apoptosis by increasing the expression of the pro-apoptotic variants of myeloid cell leukemia sequence 1 (MCL1) and B-cell lymphoma 2 (BCL2) [199].

In addition to oncogenic fusion proteins, widespread AS events occur in EwS with ribonucleoproteins that mediate oncogenic transformation via AS. A study has demonstrated that heterogeneous nuclear ribonucleoprotein M (hnRNPM) and the AS factor SRSF3 synergistically bind the intron 5 and exon 6A regions of DEAH-box helicase 9 (DHX9) to inhibit exon 6A inclusion, producing the DHX9-ΔExon6A and DHX9-Exon6A variants [200]. hnRNPM promotes the production of the non-toxic DHX9-ΔExon6A variant, stabilizing DHX9 expression and enhancing EwS cell proliferation [200]. Conversely, without hnRNPM inhibition, the DHX9-Exon6A variant is produced and rapidly degraded via nonsense-mediated decay (NMD), reducing DHX9 protein levels [200]. Thus, heightened spliceosomal activity directly contributes to drug resistance in EwS. Similarly, widespread AS programs mediated by hnRNPM have been observed following PI3K pathway inhibition in ES cells, indicating the role of AS in drug resistance [201]. In EWS-FLI1 variants, hnRNPH1 promoted the inclusion of EWS exon 8 and enhanced the expression of EWS-FLI1 variants [202]. By contrast, hnRNPH1 plays a weaker role in wild-type EWS, where exon 8 is typically excluded, forming the EWS-ΔExon8 variant [202]. This distinction highlights hnRNPH1’s specific role in regulating exon 8 splicing within the EWS-FLI1 fusion pre-mRNA, supporting EwS oncogenesis. Other scattered studies have reported AS events in EwS, such as CAPER-α scattered studies in which exon 8 is produced and rapidly degraded via nonsense [203,204]. In addition, 2 C-X-C motif chemokine receptor 4 (CXCR4) AS variants (CXCR4-3 and CXCR4-4) and a truncated death receptor 4 (DR4) variant were identified. Although CXCR4-3 shows strong ligand responsiveness, its function remains unclear while the truncated DR4 variant retains sensitivity to TRAIL (TNF-related apoptosis-inducing ligand) [205,206].

### Chondrosarcoma

**Fig. 8.**
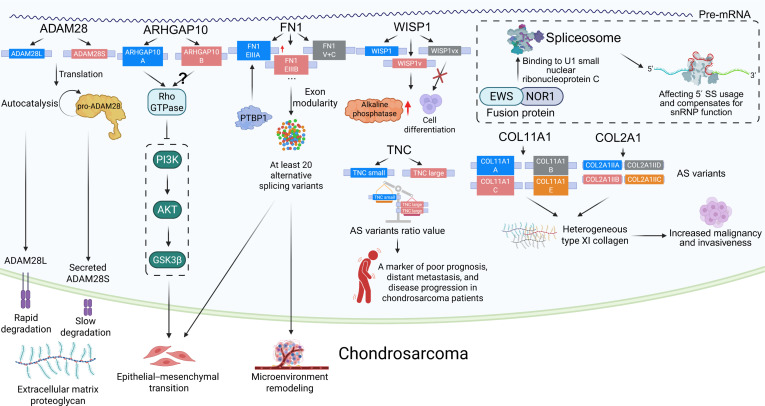
Alternative splicing and associated regulatory mechanisms in chondrosarcoma. On the left, AS variants of ADAM28 and ARHGAP10 regulate extracellular matrix degradation and PI3K-AKT-GSK3β signaling, promoting EMT and microenvironment remodeling. In the center, FN1 and WISP1 generate multiple variants that influence cell differentiation and tumor progression, while TNC variants serve as markers of poor prognosis, distant metastasis, and disease progression. On the right, COL11A1 and COL2A1 variants produce heterogeneous collagen isoforms, contributing to increased malignancy and invasiveness. At the top, EWS-NOR1 fusion proteins interact with spliceosomal components to modulate 5′ SS usage and compensate for snRNP function, further shaping AS landscapes in chondrosarcoma. ADAM28, ADAM metallopeptidase domain 28; AKT, protein kinase B; ARHGAP10, Rho GTPase activating protein 10; COL11A1, collagen type XI alpha 1 chain; COL2A1, collagen type II alpha 1 chain; EWS, Ewing sarcoma breakpoint region 1 fusion protein; FN1, fibronectin 1; GSK3β, glycogen synthase kinase 3 beta; NOR1, nuclear receptor NOR-1; PI3K, phosphoinositide 3-kinase; PTBP1, polypyrimidine tract binding protein 1; TNC, tenascin C; WISP1, WNT1 inducible signaling pathway protein 1.

CS is a slow-growing primary malignant bone tumor and the second most common malignant bone tumor after OS. AS contributes to CS progression via multiple mechanisms (Fig. 8). A study has reported that all-trans retinoic acid (ATRA) increased the release of sulfated glycosaminoglycans from the human CS cell line HCS-2/8, suggesting that ECM remodeling in CS is associated with AS [207,208]. ATRA induces the production of the long AS variant ADAM28L, rather than the short variant ADAM28S. The former exhibits greater proteoglycan-degrading activity, which is closely linked to ECM remodeling [208,209].

The CCN family influences the ECM through distinct ligands [210]. Among these, cellular communication network factor 4 (CCN4), encoded by WISP1, is a tumor growth and metastasis suppressor protein that was previously considered essential for endochondral ossification in CS [210]. However, an AS study of CCN4 challenged this view. While the full-length CCN4 protein does not affect chondrocyte differentiation, its AS variant WISP1v, which lacks exon 3, promotes differentiation and increases alkaline phosphatase levels [93]. Interestingly, HCS-2/8 cells harbor a unique AS variant of CCN4, referred to as WISP1vx, which lacks exons 3 and 4, and encodes only a truncated IGFBP (insulin-like growth factor binding protein) module protein that is incapable of influencing CS differentiation and mineralization [93]. Further studies are required to elucidate the functions of WISP1vx. The ARHGAP family encodes proteins with RhoGAP domains that regulate GTPase activity. In CS, rho GTPase-activating protein 10 (ARHGAP10) has 2 AS variants: ARHGAP10 variant A (comprising exons 1 to 23, forming a full-length protein) and ARHGAP10 variant B (comprising exons 1 to 5 and intron 5, forming a truncated protein) [211]. ARHGAP10 variant A inhibits epithelial–mesenchymal transition (EMT) by suppressing the PI3K/AKT/GSK3β (glycogen synthase kinase 3 beta) pathway; however, the role of variant B in CS remains unclear [212].

*FN1* encodes various ECM-related proteins through AS, with at least 20 variants. The skipping of exon EIIIA during chondrogenesis leads to the production of the FN1EIIIA− variant [213,214]. The EIIIA and EIIIB domains of FN1 are typically retained during embryogenesis, wound repair, and in tumors but are skipped in healthy adult tissues or more differentiated cartilage [215,216]. In rat CS cell lines, FN1 shows a variable region + connecting segment (V + C)-specific variant, low EIIIB expression, and effective inclusion of EIIIA (FN1EIIIA+), while retaining the undifferentiated chondrocytic AS pattern [214]. Intriguingly, EIIIA retention depends on approximately 200 nucleotides of the upstream intron (part of intron 32), and PTBP1 (polypyrimidine tract binding protein 1) overexpression promotes EIIIA retention [214]. Analyses of 14 osteochondrogenic tumor samples, including CS, also confirmed the inclusion of multiple variable exons, such as EIIIA and EIIIB, and the variable region, suggesting that AS facilitates CS in a dynamic remodeling state [217]. Matrix metalloproteinase 13, also known as collagenase 3, is highly expressed in tissue repair and degenerative joint diseases and is capable of degrading the cartilage matrix. It generates 4 AS variants: collagenase 3 (COL3)-APS, COL3-DEL, COL3-9B-2, and COL3-ATS [218,219]. In human CS cell lines such as SW1353, COL3-9B-2 arises from AS at intron 9, inserting the small exon 9B (related to Alu elements) that encodes a secreted protein [219]. However, whether COL3-9B-2 differs in its degradation activity from that of classical MMP-13 remains unknown. Aggrecan is a large aggregating proteoglycan in the ECM that confers elasticity to the cartilage. Its core contains G1, G2, and G3 globular domains, with the G3 domain exhibiting extensive AS [220]. Compared with normal chondrocytes, 2 additional AS variants of Aggrecan G3 are present in CS, involving partial or complete loss of epidermal growth factor-like 1 (EGF1), EGF2, and short complement repeat (SCR) domains, although their functions remain unclear [221]. Tenascin-C (TNC), a hexameric ECM protein, is highly expressed during tissue repair and in the CS. The proportion of AS variants is correlated with CS malignancy [222,223]. Compared to small TNC variants (lacking parts of the fibronectin type III domain [FN-III] repeat domain), large TNC variants (containing complete FN-III repeat domains) were more abundant in the CS cell line JJ012. As the small TNC/large TNC ratio decreases, CS progression accelerates, metastatic potential increases, and prognosis worsens [223]. The small TNC variants inhibited JJ012 adhesion in conjunction with fibronectin, whereas the large TNC variants did not [224]. Thus, TNC-AS variants serve as critical prognostic markers of CS.

Type XI collagen consists of 3 chains: α1(XI), α2(XI), and α3(XI), with multiple exons subject to selective inclusion or skipping, generating various AS variants [225]. In normal cartilage, α1(XI), α2(XI), and α3(XI) predominantly form “cartilage-type” AS variants with minimal exon inclusion [226,227]. However, in CS, α1(XI) predominantly exists as long variants (including exons 6a and 8), while α2(XI) tends to retain short “cartilage-type” variants, indicating a differentiation imbalance or cellular heterogeneity in CS [227]. Notably, the increase in long α3(XI) variants often correlates with enhanced CS malignancy and invasiveness, reflecting a reversion to an embryonic-like state [227].

Compared to normal tissues, CS expresses tumor/testis antigens, such as chondrosarcoma antigen gene (with at least 3 AS variants) and TRAG-3 (tumor-recognized antigen gene 3; with at least 4 AS variants), which enable normal expression of the melanoma antigen gene (MAGE) [228]. MAGE has become a critical target for autologous T-cell therapy, suggesting that CS could potentially serve as a candidate for T-cell therapy [229,230]. An extraskeletal myxoid CS subtype is characterized by an EWS/NOR1 fusion, producing the EWS/NOR1 fusion protein. This protein binds U1C (a U1 snRNP particle component), influencing distal 5′ SS usage in pre-mRNA, ultimately compensating for snRNP function [231,232]. This suggests that CS involves fusion proteins that directly participate in AS regulation.

### Metastatic bone cancers

**Fig. 9.**
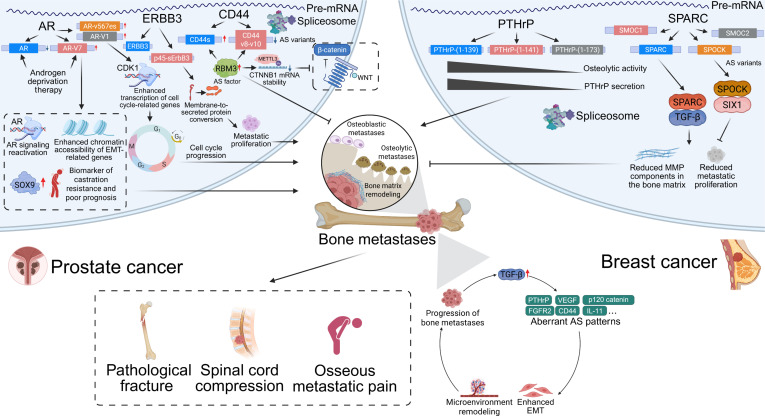
Alternative splicing and molecular mechanisms driving metastatic bone cancers. On the left, prostate cancer-associated AS variants, including AR (AR-V7 and AR-V567es), ERBB3, and CD44, enhance androgen receptor reactivation, chromatin accessibility, cell cycle progression, and metastatic proliferation, contributing to castration resistance and poor prognosis. On the right, breast cancer-related AS events in PTHrP, SPARC, and CD44 modulate osteolytic activity, extracellular matrix remodeling, and metastatic proliferation. In the center, aberrant AS patterns converge on the bone microenvironment to promote osteoblastic and osteolytic metastases, driven by TGF-β signaling, EMT, and microenvironment remodeling, leading to clinical complications such as pathological fractures, spinal cord compression, and metastatic bone pain. AR, androgen receptor; CD44, CD44 molecule; CDK1, cyclin-dependent kinase 1; ERBB3, Erb-B2 receptor tyrosine kinase 3; FGFR3, fibroblast growth factor receptor 3; IL11, interleukin 11; PTHrP, parathyroid hormone-related protein; RBM3, RNA binding motif protein 3; SIX1, SIX homeobox 1; SMOC1, SPARC-related modular calcium binding 1; SMOC2, SPARC-related modular calcium binding 2; SPARC, secreted protein acidic and cysteine rich; SPOCK, SPARC/osteonectin, cwcv, and kazal-like domains proteoglycan; TGFβ, transforming growth factor beta; VEGFA, vascular endothelial growth factor A.

Bone metastases are common and can be classified as osteoblastic and osteolytic lesions based on their distinct characteristics (Fig. 9). These metastases often lead to complications, such as pathological fractures, spinal cord compression, and bone pain [233]. In lung cancer, truncated AS variants of ADAM8 exhibit structural alterations that expose integrin-binding domains, enhancing interaction with integrins [234]. This increases osteoclast activity, drives osteolytic destruction, and promotes the development and expansion of bone metastases [234]. Prostate cancer (PCa) frequently metastasizes to bone, and PCa cells often employ androgen receptor (AR) AS to evade androgen deprivation therapy or AR signaling inhibitors [235]. The AR variant AR-V7, which lacks the ligand-binding domain (corresponding to exons 4 to 8) but retains the constitutively active N-terminal domain, cannot be targeted by AR inhibitors. This variant transcriptionally activates AR signaling and is considered a key driver of resistance [236–238]. In castration-resistant PCa (CRPC), bone metastases exhibit increased aggressiveness and osteoblastic lesions in CRPC. AR-V7 enhances PCa bone metastasis by increasing the chromatin accessibility of EMT-related genes and up-regulating the transcription of the metastasis driver gene SOX9 (sex-determining region Y-box 9), highlighting the critical role of AS in bone metastasis [239]. AR-V7 has been implicated in shaping the immunosuppressive phenotype of PCa. Prophylactic vaccination targeting AR-V7 induces robust antitumor immune responses in preclinical models, but its efficacy is reduced in immunosuppressive settings where local CD8^+^ T-cell exclusion may counteract vaccine effects [240]. Transcriptomic analyses show that AR-V7 overexpression down-regulates pathways such as “leukocyte migration” and “chemokine-mediated signaling pathway”, and similar suppression of immune-related gene programs has been reported in breast cancer [240,241]. These findings indicate that AR-V7 may promote immune evasion through direct or indirect mechanisms. In addition, other AR variants, such as AR-V1 and AR-v567es, are expressed in PCa bone metastases. AR-V1 is composed of exons 1 to 3 and the cryptic exon CE1, whereas AR-v567es retains exons 1 to 4 but lacks exons 5 to 7, resulting in a frameshift and premature stop codon after the 10th amino acid of exon 8, forming a truncated protein [242]. These variants also lack the ligand-binding domain but exhibit constitutive activity, directly activating downstream AR transcription programs, particularly cell cycle-related genes such as cyclin-dependent kinase 1 (CDK1), thereby promoting the progression of bone metastases [242,243]. The CRPC-specific Erb-B2 receptor tyrosine kinase 3 (ERBB3) AS variant p45-sErbB3 lacks the transmembrane domain and the C-terminal sequences, transforming ERBB3 from a transmembrane protein into a paracrine factor [244]. p45-sErbB3 is closely associated with CRPC progression, stimulating osteoblast proliferation and differentiation and promoting the secretion of bone sialoprotein and other factors in the bone microenvironment, contributing to PCa bone metastases [245]. Cathepsin Z (CTSZ) mediates the degradation of the AS factor TRA2A via the proteasomal pathway, thereby promoting the expression and secretion of specific IL-32 variants and lifting the molecular suppression of macrophage infiltration [246]. This process activates the ITGA5/PI3K signaling axis in macrophages, markedly enhancing the recruitment of M2 macrophages and ultimately accelerating the metastatic progression of PCa [246].

Certain AS factors influence PCa bone metastasis. For instance, overexpression of RBM3 (RNA binding motif protein 3) and other factors in the bone microenvironment contributes to the regulation of cell cycle–related genes such as CDK1, thereby promoting the metastatic potential [247]. CD44, composed of 10 constant exons and 10 variable exons (v1 to v10) inserted between exons 5 and 16, generates distinct CD44 variants [248]. RBM3 suppresses the CD44v8 to v10 variant and increases the CD44s (standard CD44, lacking variable exons) variant, reducing PCa invasiveness and metastatic ability and offering a new avenue for addressing PCa bone metastases [249]. Moreover, CD44 variants serve as biomarkers of tumor recurrence and progression in ampullary adenocarcinoma, particularly CD44v3-10 and CD44v6-10 [250]. In metastatic prostate adenocarcinoma, the AS factor HSPB1 (heat shock protein family B member 1) potentially regulates AS events in genes, such as SRC, EGFR (epidermal growth factor receptor), MAPT (microtubule-associated protein tau), APP (amyloid precursor protein), and PRKCA (protein kinase C alpha), thereby contributing to bone metastasis [251]. Interestingly, intronic BP site switching may play a role in PCa. In normal bone tissue versus metastatic PCa, BP site switching was observed in the first intron of HBB (hemoglobin subunit beta), potentially disrupting U2 snRNA binding [252]. This suggests that tumor-specific AS environments guide the selection of intronic BP sites. The prostate-specific membrane antigen produces at least 3 AS variants: prostate-specific membrane (PSM), PSM-C, and PSM-D. However, only PSM-D is expressed at higher levels in lymph nodes and bone metastases than in primary tumors, indicating its specific role in PCa metastasis [253].

Bladder cancer (BLCA), a malignant lesion originating from bladder epithelial cells, is the second most common genitourinary malignancy that frequently metastasizes to the bones. In patients with BLCA and bone metastases, the AS factor JUP (junction plakoglobin) was identified as a regulator of the AP of SMOX (spermine oxidase) and the exon-skipping event of ITGB4 (integrin subunit beta 4), which influences BLCA metastasis [254]. Additionally, in mesothelioma, the AS factors HSPA1A (heat shock protein family A member 1A) and DDX3Y (DEAD-box helicase Y) regulate the AS of SNX5 (sorting nexin 5), affecting immune evasion and bone metastasis [255].

Osteopontin (OPN) facilitates bone metastasis in breast cancer and PCa and generates 3 AS variants: OPN-a (full-length), OPN-b (lacking exon 5), and OPN-c (lacking exon 4) [256,257]. Overexpression of OPN-a promotes the adhesion of non-small cell lung cancer (NSCLC) cells to bone tissue via αvβ3 integrin, and blocking αvβ3 integrin reduces adhesion [258]. Compared to adjacent tissues, OPN-b is highly expressed in soft tissue sarcomas and is associated with tumor staging, whereas OPN-c is correlated with tumor stage and lymph node metastasis in soft tissue sarcomas [257]. However, the roles of OPN-b and OPN-c in bone metastasis remain unclear.

In breast cancer bone metastases, parathyroid hormone-related protein (PTHrP) facilitates osteolytic destruction via 3 AS variants: PTHrP-1-139, PTHrP-1-141, and PTHrP-1-173 [259]. PTHrP-1-139, which includes exons 6 and 7, exhibits high secretion and osteolytic activity, leading to hypercalcemia. PTHrP-1-141 includes exons 6, 7, and 8, which have lower secretion and osteolytic activity [85,260]. PTHrP-1-173, the longest variant containing exons 6 to 9, exhibits minimal secretion and osteolytic activity [85]. These AS variants selectively promote osteoclast formation and bone resorption, providing a growth advantage for breast cancer bone metastases, and offering a novel evaluation metric for patients with breast cancer bone metastases.

**Fig. 10.**
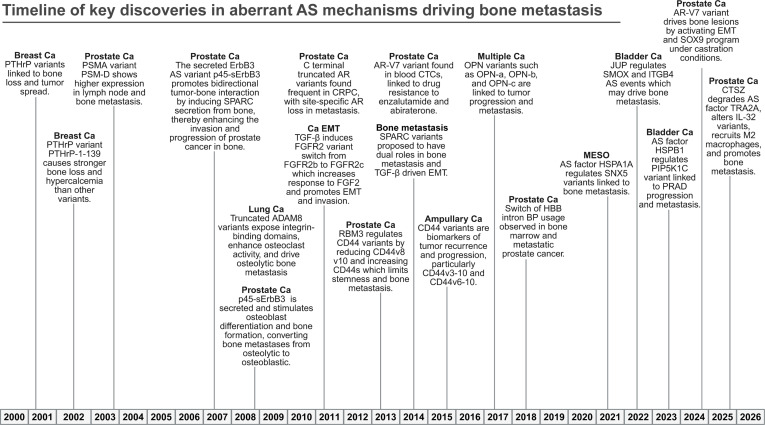
Timeline of key discoveries in aberrant AS mechanisms driving bone metastasis. AR, androgen receptor; AR-V7, androgen receptor variant 7; BP, branch point; Ca, cancer; CD44, CD44 molecule; CTSZ, cathepsin Z; EMT, epithelial–mesenchymal transition; FGFR2, fibroblast growth factor receptor 2; HBB, hemoglobin subunit beta; HSPA1A, heat shock protein family A member 1A; HSPB1, heat shock protein family B member 1; IL-32, interleukin-32; ITGB4, integrin subunit beta 4; ITGA5, integrin subunit alpha 5; JUP, junction plakoglobin; MESO, mesothelioma; OPN, osteopontin; PCa, prostate cancer; PIP5K1C, phosphatidylinositol-4-phosphate 5-kinase type 1 gamma; PSM, prostate-specific membrane antigen; PTHrP, parathyroid hormone-related protein; RBM3, RNA binding motif protein 3; RANKL, receptor activator of nuclear factor kappa-B ligand; SMOX, spermine oxidase; SNX5, sorting nexin 5; SPARC, secreted protein acidic and rich in cysteine; TGF-β, transforming growth factor beta.

Cysteine-rich secreted proteins (secreted protein acidic and rich in cysteine [SPARC]) enhance bone metastasis and EMT in glioblastoma, melanoma, and PCa by modulating cell–matrix interactions [261]. However, SPARC suppresses proliferation, migration, and bone metastasis in breast cancer, as validated in gene-knockout mice [261–263]. SPARC undergoes AS to produce at least 5 variants (SMOC1 [SPARC-related modular calcium-binding protein 1], SMOC2 [SPARC-related modular calcium-binding protein 2], SPOCK [SPARC-related osteonectin-like protein], follistatin-like 1, and SPARC-like-1), with N- or C-terminal domain deletions altering integrin and growth factor binding, exhibiting effects opposite to those of full-length SPARC in the adhesion, migration, and EMT processes [264]. For example, in breast cancer, SPOCK interacts with SIX1 to promote EMT and cell proliferation via the AKT/mTOR (mechanistic target of rapamycin) pathway [265]. Notably, SPARC variant generation and function are regulated by key signaling molecules such as TGF-β in bone metastases [264,266]. As TGF-β accumulates in the bone matrix and is activated during osteolytic destruction in bone metastases, it forms a positive feedback loop with signals such as PTHrP, VEGF, and interleukin-11, enhancing the invasiveness and EMT levels of bone metastases [267]. Furthermore, TGF-β directly regulates CD44, p120 catenin, and fibroblast growth factor receptor 2 (FGFR2) [267]. Due to mutually exclusive exon IIIb and exon IIIc usage, FGFR2 generates 2 AS variants, FGFR2b and FGFR2c, with FGFR2b primarily expressed in epithelial cells and FGFR2c in mesenchymal cells [268]. TGF-β stimulation increases FGFR2c levels, enhancing sensitivity to fibroblast growth factor 2 and promoting EMT, suggesting that inhibiting TGF-β signaling could mitigate tumor bone metastases through AS modulation [269]. The timeline highlights the key discoveries of aberrant AS in bone metastasis over the past 20 years (Fig. 10).

### Physiological and pathological AS in bone

We summarized the characteristics of AS in bone tissue under physiological and pathological conditions, which can be compared across several dimensions (Table 3). In terms of shared features, both states rely on core splicing regulators, such as serine/arginine-rich splicing factors (e.g., SRSF1 and SRSF3) and hnRNPs, which coordinate exon selection and splice site recognition. In addition, the predominant types of splicing events—ES, IR, and MXE—are observed in both bone development and tumor progression. Third, as a post-transcriptional regulatory mechanism, AS dynamically modulates gene expression in both contexts, influencing osteogenic differentiation, matrix remodeling, and cellular responses to external stimuli.

**Table 3.** Comparative characteristics of AS in bone under physiological and pathological conditions

	AS under physiological conditions	AS under pathological conditions
Functional purpose	Regulates bone development, bone formation and resorption balance, and mechanical stimulation responses	Promotes tumor progression, immune evasion, therapy resistance, and metastatic dissemination
Cell types	Osteoblasts, osteoclasts, chondrocytes, and mesenchymal stem cells	Tumor cells in bone, especially osteosarcoma cells, chondrosarcoma cells, and bone-metastatic tumor cells
Representative AS factors	SRSF1/5, HNRNPLL, CPEB4, FLNB, DDX5, etc.	SRSF1/3/6, YBX1, hnRNPM/H1, METTL3, GEMIN5, etc.
AS event types	Regulates developmental transitions primarily through ES and MXE	Drives tumor progression via excessive ED, IR, A5SS, and A3SS
Functional impact of AS variants	Determines cell fate decisions and influences extracellular matrix composition	Alters protein activity, initiates oncogenic programs, promotes immune escape and angiogenesis
Bone remodeling	RANKL-AS, RANK-c, and Periostin-4 regulate bone remodeling via AS.	Tumor-activated RANKL up-regulation and shedding, leading to bone degradation (e.g., PTHrP-139)
Downstream pathways	Mainly affects bone formation via Wnt, BMP, and TGF-β signaling pathways	Mainly drives tumor progression via MAPK, PI3K-AKT, EMT, immune suppression, and metabolic reprogramming
Clinical relevance	Still underexplored; some AS variants show potential as developmental or mechanoresponsive biomarkers	Potential prognostic markers (e.g., VEGF165b, AR-V7, and Hdmx-S) or therapeutic targets (e.g., IL-24 variants)

In bone tissue, AS is far more than simply “cutting out the exon”. AS constructs distinct transcriptomic landscapes under physiological and pathological states. In homeostatic conditions, AS functions like a precise engineer, regulating the differentiation rhythm of bone cells, mediating responses to mechanical stimuli, and maintaining dynamic remodeling of the bone matrix. Upon transition into a pathological state, the role of AS shifts subtly but decisively, becoming a driver of tumor progression. In OS, ES, and bone metastases, AS ceases to be a tool for homeostatic regulation and instead participates in amplifying oncogenic signaling, promoting therapeutic resistance, and enabling immune evasion. By generating splice variants that promote angiogenesis, EMT, migration, or resistance to apoptosis, AS directly facilitates tumor cell adaptation to complex microenvironments and enables distant dissemination. This view is further supported by studies of AS in other malignancies [11,16,270]. Of note, certain AS events themselves serve as functional boundaries between physiological and pathological states. For example, AS of VEGFA produces VEGF165 and VEGF165b isoforms, with the former promoting angiogenesis and the latter acting as an endogenous inhibitor. Up-regulation of YBX1 disrupts this balance by favoring VEGF165 expression, thereby facilitating tumor progression. Similarly, AS of oncogenes such as *IGF1* and *TP53* can shift their functional roles from promoting osteogenesis to driving tumorigenesis, depending on the splicing pattern.

Although AS-related research in other pathological bone conditions (e.g., osteoporosis, OA, and hereditary bone diseases) is relatively limited, we have summarized the key findings in these fields. First, in osteoporosis-related AS research, conditional knockout of NIBAN2 in male mouse osteoblasts does not affect overall body weight or developmental processes, but leads to insufficient femoral mineralization and an osteoporotic phenotype [271]. Mechanistically, NIBAN2 stabilizes the binding of core components of the HNRNPU splicing complex (e.g., DDX39B and RBMX) to promote retention of RUNX2 exon 6 (containing the nuclear localization sequence), thereby generating a functional RUNX2 protein capable of nuclear translocation and transcriptional activation of osteogenic differentiation [271]. In addition, with aging, the expression of the AS factor YBX1 is reduced in mouse BMSCs, a change closely associated with decreased bone mass and enhanced adipogenic differentiation [272]. Conditional knockout of Ybx1 using Prx1-Cre in mice accelerates bone loss, whereas Ybx1 overexpression in BMSCs promotes osteogenesis [272]. Mechanistically, YBX1 deficiency induces aberrant AS of multiple genes associated with osteogenesis and aging (e.g., Fn1, Sp7, Spp1, Sirt2, and Nrp2), thereby accelerating BMSC senescence and driving adipogenic lineage commitment [272]. Moreover, glucocorticoids, through the HDAC4-FOXC1 epigenetic axis, down-regulate SRSF1 expression in osteoblasts, resulting in up-regulation of Bmf v2 and v3 AS variants, which enhances osteoblast apoptosis [273]. This finding highlights the critical role of SRSF1 in glucocorticoid-induced osteoporosis and osteonecrosis [273]. In human cohorts with fractures, genome-wide association studies combined with transcriptomic analyses have identified candidate AS genes associated with osteoporosis, including USP48, MAEA, and KLHL8 [274]. These statistical associations still require further functional studies to confirm causality and elucidate potential mechanisms.

Secondly, AS has also been implicated in OA. Differential AS events have been identified between OA and healthy meniscus tissues, involving apoptosis-related genes such as *BCL2L13*, *XAF1*, and *NF2*, as well as OA progression-related genes including *IL-16* and *PRG4* [275–277]. In a study of 101 primary chondrocytes (FN-f stimulation vs. PBS control), a total of 7,188 splicing quantitative trait loci (sQTLs) were mapped [278]. Colocalization of sQTLs with OA GWAS signals further identified 6 potential OA-associated AS genes, namely, *PBRM1*, *WWP2*, *COLGALT2*, *GCAT*, *HMGN1*, and *RNF144B* [278], providing a foundation for future mechanistic studies and targeted interventions in OA. The AS factor DDX5 is down-regulated during OA progression, leading to increased proportions of full-length FN1 and PLOD2 variants. This shift drives cartilage fibrosis and degradation by up-regulating fibrosis-related genes (e.g., *COL2A1*, *COL1A1*, and *COL1A2*) and matrix-degrading genes (e.g., *MMP13* and *NOS2*) [106]. Conditional knockout of Ddx5 in chondrocytes demonstrated that targeted regulation of Fn1 and Plod2 can partially rescue the OA phenotype, suggesting that restoration of DDX5-mediated AS balance may represent a novel therapeutic strategy for OA [106]. In a post-traumatic OA mouse model, the CALCA gene was shown to generate 2 distinct AS products: the procalcitonin/calcitonin (PCT/CT) variant, which attenuated cartilage degeneration and subchondral bone loss but exacerbated synovial inflammation, and the αCGRP variant, which promoted osteophyte formation [279]. These findings highlight that AS products derived from the same gene may exert divergent or even opposing functions during OA progression [279].

Finally, in the field of hereditary bone diseases, approximately 90% of osteogenesis imperfecta (OI) cases are caused by mutations in the type I collagen α-chain genes (collagen type I alpha 1 chain/collagen type I alpha 2 chain [COL1A1/COL1A2]), leading to either quantitative defects (reduced synthesis) or qualitative defects (structural abnormalities) of type I collagen [280,281]. One familial study identified a 562-bp deletion spanning the end of exon 34 to the middle of intron 36 in COL1A1, with the mutant allele undergoing aberrant splicing to generate 3 transcripts (IR, multi-exon skipping, and cryptic donor usage). As a result, some in-frame transcripts failed to produce stable α1(I) chains, accompanied by an abnormal increase in type III collagen [282]. This AS defect was consistent with the severe type III OI phenotype observed in patients [282]. Another study reported that a COL1A1 donor splice site mutation (c.1155+1G>C) caused exon 17 skipping, thereby disrupting α1(I) chain integrity and producing a typical autosomal dominant OI phenotype [283]. Similarly, a donor splice site mutation in COL1A2 intron 46 (IVS46+2T>C) activated an upstream cryptic splice site, leading to a 17-bp deletion, frameshift, and premature termination. This led to the loss of the α2(I) chain and the exclusive formation of unstable α1(I) homotrimers, consequently giving rise to a dominant OI phenotype [284]. In addition, a splice site mutation in intron 3 of TMEM38B (c.455-7T>G) was identified in Chinese Han OI patients, which impaired TRIC-B ion channel function and contributed to autosomal recessive OI [285]. Collectively, these findings highlight AS as an important molecular mechanism in OI pathogenesis and a potential therapeutic target. Beyond OI, mutations in COL2A1 affecting exon 12, exon 21, and the intron 20/exon 21 junction have been closely associated with chondrodysplasias [286–289]. Splice site mutations in PHEX are closely linked to X-linked hypophosphatemia [290–293], while splice alterations in TCIRG1 are associated with osteopetrosis [294–297].

In summary, AS under physiological and pathological conditions displays markedly different characteristics, providing new opportunities for future research (Table 4). For example, by integrating single-cell sequencing and spatial transcriptomics, it will be possible to systematically dissect the AS patterns of osteoblasts, chondrocytes, and osteoclasts under mechanical stimulation, thereby constructing an atlas of mechanically regulated AS. In this direction, several groups have already developed dedicated computational algorithms to decode the complexity of AS networks [298–300]. Furthermore, exploring the AS networks involved in the crosstalk between immunity and bone metabolism is another promising avenue. Osteoimmunology has already produced many interesting findings, such as T cells and B cells directly regulating osteoclastogenesis through the RANKL–RANK–OPG axis [301,302]. However, the role of AS in osteoimmunology remains unexplored, which represents a potential innovation point. Finally, whether AS variants or AS factors can serve as therapeutic targets could first be prospectively validated in patient cohorts using liquid biopsy [303]. After identifying patient subgroups dependent on specific AS events, targeted drug development could then be pursued, which holds promise to significantly improve the success rate of clinical trials [303,304].

**Table 4.** Comparative roles of AS in bone biology, tumor biology, and other diseases in physiological and pathological contexts

	Bone biology	Tumor progression	Other diseases
Core role	Regulates cell differentiation and homeostasis	Acquisition of malignant phenotypes, promoting adaptation and proliferation	Regulation of bone mass, cartilage integrity, and matrix stability
Major functions	1.Regulation of cell differentiation: AS modulates the differentiation and function of osteoblasts (e.g., via RUNX2 protein), osteoclasts (e.g., RANKL variant), and chondrocytes (e.g., COL2A1 variant). 2. Response to mechanical stimulation: For example, the MGF variant and SRSF1 protein convert mechanical signals into osteometabolic signals. 3. Maintenance of bone matrix homeostasis: Regulation of collagen and other matrix protein variants influences bone quality.	1. Accelerated proliferation: Generation of pro-proliferative or anti-apoptotic variants (e.g., PKM2 and BCL-XL). 2. Invasion and metastasis: Variants of adhesion, cytoskeleton, and EMT-related genes are affected, enhancing cell migratory capacity. 3. Angiogenesis: Selection of variants that favor tumor survival (e.g., increased VEGF165 variant). 4. Therapy resistance: Production of drug-resistant variants (e.g., AR-V7 against anti-androgen therapy).	1. Neurological disorders: Neuron-specific aberrant AS causes protein dysfunction (e.g., in SMA, AS defects in SMN2 result in insufficient functional SMN protein). 2. Osteoporosis: AS of RUNX2, YBX1, and SRSF1 affects bone mass and the balance between osteogenic and adipogenic differentiation. 3. Osteoarthritis: Variants of FN1, PLOD2, and CALCA regulate cartilage degradation and osteophyte formation. 4. Hereditary bone diseases: Splicing mutations in COL1A1/2, TMEM38B, PHEX, and TCIRG1 directly cause disease.
Primary drivers	Primarily physiological signals. 1. Hormones (e.g., parathyroid hormone), cytokines, mechanical stimulation.	Primarily genetic mutations and aberrant activation. 1. Mutations in trans-acting factors: Mutations in splicing factors such as SF3B1 and SRSF2. 2. Mutations in cis-regulatory elements: Creation or disruption of SS in oncogenes or tumor suppressor genes. 3. Dysregulated signaling pathways: Abnormal activation of kinase pathways (e.g., SRPK) that regulate splicing factors.	Hereditary mutations or age/environment-associated dysregulation of AS. 1. Direct mutations: For example, deletion of the SMN1 gene. 2. Age-related decline in regulation (e.g., YBX1), drug effects (e.g., glucocorticoids), and congenital mutations (COL1A1/2, TMEM38B, PHEX, and TCIRG1).
Features	Plasticity: AS acts as a molecular switch that supports bone to remodel and repair itself in response to environmental changes.	Heterogeneity and adaptability: AS serves as an evolutionary tool for tumors, generating internal diversity and enabling escape from therapeutic pressure.	Specificity: AS alterations in bone diseases converge on key structural or metabolic genes (e.g., collagen, channels, and metabolic enzymes).
Commonalities	1. A universal regulator of core cellular processes: Across all fields, AS finely tunes cell proliferation, differentiation, metabolism, and death by generating multiple protein variants from a single gene. 2. Responsiveness to environmental signals: Whether in bone, tumors, or other tissues, AS is a dynamic process that responds to external (e.g., drugs and mechanical forces) and internal (e.g., aging and mutations) signals. 3. A repository of diagnostic and therapeutic targets: Aberrant AS variants serve as disease-specific biomarkers, while the AS process itself represents an emerging therapeutic target.		

## Advances in AS-Targeted Therapeutics

Following the previous sections that highlighted the critical roles of AS in bone biology and tumor progression, we now turn our attention to its clinical relevance and translational potential. Targeted therapies developed based on AS mechanisms can be categorized into 3 approaches: targeting abnormal AS variants, targeting cis-regulatory elements of pre-mRNA, and targeting AS factors [305–307]. Targeting abnormal AS variants or cis-regulatory elements entails the precise delivery of exogenous agents to correct redundant or pathogenic AS variant functions in defined tissues or organs. Potential strategies include the use of ASO, gene editing, RNA aptamers, siRNA, and shRNA, which offer the advantage of high specificity and enable personalized treatment [306]. However, a major limitation is that nucleic acid drugs such as ASO typically require administration via injection and face difficulty crossing the blood–brain barrier. In addition, unmodified ASOs are unstable in vivo, prone to degradation by nucleases, and trigger immune responses, thus necessitating chemical modifications to enhance stability and reduce immunogenicity [308,309]. Targeting AS factors essentially involves activating or inhibiting specific protein activities through small-molecule drugs to reverse abnormal splicing patterns [310,311]. Small-molecule therapy can be administered orally or intravenously, providing better bioavailability and widespread systemic distribution, but the main drawback lies in its limited tissue specificity [312]. A single AS factor is often involved in the AS of multiple genes, which could result in non-specific effects and potential side effects, thereby increasing the complexity of predicting drug responses [313,314]. We have summarized AS-related targeted therapeutics that are either approved or currently undergoing clinical trials, and explored their potential applications in bone-related diseases (Table 5).

**Table 5.** AS-modulating therapeutic agents and prospects in bone disease

Therapeutic agent	Mechanism	Disease target	Development stage	Potential application in bone-related diseases	References
Nusinersen (Spinraza)	ASO corrects SMN2 gene splicing errors, increases functional SMN protein expression.	SMA	FDA approved	Potential applications in musculoskeletal degenerative diseases	[320,321]
Risdiplam (Evrysdi)	Small molecules modulate SMN2 AS to promote functional SMN protein production.	SMA	FDA approved	Similar to Nusinersen; potential applications in musculoskeletal diseases	[322,324]
Denosumab	Neutralize sRANKL and membrane RANKL, blocking RANK engagement and osteoclastogenesis	Osteoporosis and giant cell tumor	FDA approved	Osteoporosis and giant cell tumor of bone. Prevent skeletal-related events in bone metastases/multiple myeloma	[381,382]
Branaplam	Small molecules regulate splicing to increase functional SMN2 transcripts.	SMA, Huntington’s disease	Clinical trial stage	No current direct bone-related applications; suggests potential for small molecules in bone AS-related disorders	[327,328]
E7107	SF3B1 inhibitor that disrupts spliceosome assembly to suppress abnormal splicing in tumor cells	Solid tumors and leukemia	Clinical trial stage	Potential application in bone marrow tumors and leukemia-associated bone pathologies	[329,330,332]
Sudemycin family	Small molecules targeting SF3B1 to disrupt spliceosome function and tumor-related splicing events	Leukemia and other malignancies	Preclinical research stage	Potential for use in multiple myeloma and bone metastasis tumors	[333,383]
H3B-8800	Small molecule targeting SF3B spliceosome complex, selectively modulates abnormal AS events	MDS and AML	Clinical trial stage	Direct application prospects in MDS	[335,384]
PRMT5 inhibitors	Inhibits PRMT5-mediated arginine methylation, disrupts spliceosome assembly and DNA repair, induces apoptosis	MDS, CMML, and solid tumors	Clinical trial stage	Under physiological conditions, PRMT5 inhibition promotes osteoblast differentiation, restricts excessive proliferation, and influences chondrocyte development to maintain bone homeostasis; in pathological contexts, it reduces glycolysis, suppresses tumor growth, and impairs DNA repair, demonstrating therapeutic potential in bone tumors.	[342,344]
PKM ASO	ASO regulates PKM AS, promotes conversion from PKM2 to PKM1.	Liver cancer and glioblastoma	Preclinical research stage	Influence bone tumor treatment strategies through metabolic pathways	[337,340]
SRPK inhibitors	Block SRPK to affect SR protein activity and AS process	Leukemia and neovascular diseases	Preclinical research stage	Possible applications in bone tumors and bone metastases, especially angiogenesis-related bone tumors	[354,360]

A classic example comes from the development of therapeutic strategies for SMA. SMA is primarily caused by deletions or mutations in the SMN1 gene, leading to a deficiency of SMN protein [315]. Fortunately, a homologous gene, SMN2, exists in the human genome, but due to exon 7 exclusion, SMN2 predominantly generates unstable or non-functional protein [315]. Nusinersen is an 18-nucleotide ASO that targets the ISS-N1 region downstream of intron 7 in SMN2 pre-mRNA, a cis-acting element that inhibits exon 7 inclusion [316]. By masking this region, Nusinersen promotes exon 7 retention, increases the proportion of full-length SMN2 mRNA, and elevates the expression of functional SMN protein, thereby partially compensating for the deficiency of SMN1 [317–319]. Among patients treated with Nusinersen, 51% of infants achieved motor developmental milestones (e.g., independent sitting, rolling, and standing) post-treatment, compared to 0% in the control group, and also exhibited prolonged survival without ventilatory support, leading to a substantial improvement in quality of life for patients with SMA [320,321]. Risdiplam, a diazine derivative, is an orally administered small-molecule splicing modulator that selectively targets 2 sites in SMN2 pre-mRNA, namely, the exon 7 ESE and the intron 7 5′ SS, in order to promote exon 7 inclusion and thereby increase functional SMN protein production to alleviate SMA symptoms [322,323]. Clinical studies of Risdiplam in patients with SMA types 1, 2, and 3, including infants, children, and young adults, demonstrated improvements in motor function, increased survival, and favorable tolerability, offering an effective oral therapeutic option for SMA patients across various types and age groups [324,325]. Another splicing modulator, Branaplam, had entered clinical studies for SMA and Huntington’s disease but was discontinued due to safety concerns [326,327]. Mechanistically, Branaplam modulates the 5′ SS through a sequential 3-step binding process: U1-C first reversibly binds U1 snRNP, then recognizes aberrant 5′ SS featuring a -1A bulge, ultimately forming a Branaplam-U1 snRNP-5′ SS RNA complex that stabilizes splice site recognition and promotes exon retention [328]. Taken together, insights gained from the therapeutic development of SMA have profound implications for splicing-targeted strategies. First, precise identification of pathogenic AS variants is foundational to the success of ASO-based therapy. Second, promoting exon retention represents a mechanistically clear and clinically validated pre-mRNA intervention strategy. Third, targeting AS factors offers broad regulatory potential but necessitates careful evaluation of off-target splicing alterations and associated safety risks.

E7107 is the first small-molecule spliceosome inhibitor to enter clinical trials. It targets the SF3B1 subunit and disrupts the recognition of the branch point in pre-mRNA by the U2 snRNP, thereby blocking spliceosome assembly and inducing NMD and reductions in mature mRNA levels [329–331]. In a Phase I trial for advanced solid tumors, although 8 patients exhibited stable disease following E7107 treatment, the trial was terminated due to 2 cases of E7107-associated irreversible vision loss [329]. Subsequent preclinical studies found that E7107 strongly induced apoptosis in uveal melanoma and pancreatic ductal adenocarcinoma cell lines, although clinical trial data remain unavailable [330,332]. Similarly, newer SF3B1-targeting inhibitors have emerged, including the Sudemycin family and H3B-8800, which demonstrate stronger splicing interference and cytotoxicity in tumor cells with comparatively milder effects on normal cells, indicating potential clinical applicability [333,334]. Among them, the Phase I clinical trial of H3B-8800 observed alleviation of red blood cell transfusion dependence in a subset of myelodysplastic syndrome (MDS) patients harboring SF3B1 missense mutations. However, due to the absence of objectively defined clinical responses, further clinical development was discontinued [335]. In summary, experience from the development of SF3B1 inhibitors offers several important insights. Although AS factors are often hyperactivated in tumors, their ubiquitous expression across normal tissues poses a substantial risk of toxicity, particularly to functionally sensitive organs such as the eyes. Moreover, while mutations in splicing factors confer increased drug sensitivity, such genetic alterations alone are insufficient to achieve complete tumor eradication. These findings underscore the necessity for splicing-targeted therapies to account for tissue-specific regulatory dynamics and the functional consequences of modulating distinct AS events.

The AS variant of the *PKM* gene represents a representative case of ASO application. In human PKM pre-mRNA, exons 9 and 10 are mutually exclusive, generating 2 AS variants: PKM1 (containing exon 9) and PKM2 (containing exon 10) [336]. Up-regulation of PKM2 promotes aerobic glycolysis, providing substrates for anabolic processes such as lipid and nucleotide synthesis, while also activating transcription factors including HIF-1α, β-catenin, and STAT3, thereby enhancing cellular proliferative capacity. This mechanism plays a critical role in the progression of inflammatory bone diseases and OS [337–339]. A PKM ASO modified with constrained ethyl (cEt) bases and optimized with a phosphorothioate backbone effectively blocks a cis-regulatory element that promotes exon 10 inclusion in PKM pre-mRNA, inducing a controlled switch from PKM2 to PKM1 splicing. This approach has been shown to suppress the progression of hepatocellular carcinoma and glioblastoma [336,340]. These findings suggest that in bone-related diseases characterized by aberrant PKM2 up-regulation, PKM ASO could represent a promising therapeutic strategy, though its efficacy remains to be further validated in clinical trials. Additional ASO targets, including BCL-X, MNK2, MDM4, and PD-L1, have been extensively discussed in previous reviews [305].

Protein arginine methyltransferase 5 (PRMT5) regulates pre-mRNA splicing by methylating AS factors, including members of the Sm and SR protein families [341]. At least 12 PRMT5-targeting compounds are undergoing clinical investigation [342]. Mechanistically, in diseases harboring AS factor mutations (such as MDS and chronic myelomonocytic leukemia), tumor cells become dependent on PRMT5 to maintain a “barely stable” splicing system; thus, PRMT5 inhibition induces synthetic lethality, selectively triggering tumor cell death [343,344]. Furthermore, homozygous deletion of methylthioadenosine phosphorylase (MTAP) leads to the accumulation of MTA, which renders cells dependent on the residual activity of PRMT5, thereby disrupting AS and exposing a novel therapeutic vulnerability [345,346]. PRMT5 inhibitors have demonstrated encouraging results in clinical trials for various solid tumors, including NSCLC, pancreatic cancer, biliary tract cancer, and esophageal carcinoma. Moreover, PRMT5 regulates the AS of key DNA repair factors TIP60 and SUV4-20H2, affecting their enzymatic activities and chromatin-modifying functions, thereby participating in the homologous recombination (HR) repair pathway [347]. Based on this, PRMT5 inhibition can induce an “HR deficiency” phenotype, and its combination with PARP inhibitors is expected to induce synthetic lethality [348]. Inhibition of PRMT5 also enhances osteogenic differentiation of mesenchymal stromal cells while reducing their proliferative capacity [347,349,350]. Additionally, PRMT5 inhibition suppresses osteoclast differentiation and impairs chondrocyte development, suggesting a critical role for PRMT5 in skeletal development [351,352]. In soft tissue sarcomas, including OS, PRMT5 inhibition reduces glycolytic rates and slows tumor cell proliferation [353]. Collectively, evidence highlights PRMT5 inhibitors as promising therapeutic targets for bone tumors, particularly in tumor subtypes exhibiting high dependency on PRMT5 and harboring specific splicing factor mutations. In addition, considering the essential function of PRMT5 in maintaining HR repair, its inhibition in combination with PARP inhibitors offer a novel approach for the precision treatment of bone malignancies.

Serine/arginine-rich protein-specific kinase (SRPK), as kinases, activate SR proteins through phosphorylation, alter their nuclear and cytoplasmic localization, promote spliceosome assembly, and participate in the pathogenesis of NSCLC, triple-negative breast cancer, leukemia, and gliomas [354–358]. In leukemia, SRPK enhances the splicing regulatory capacity of SRSF1 on cyclin A1 pre-mRNA by phosphorylating SRSF1 at S422, thereby accelerating cell cycle progression and promoting tumor cell proliferation [357]. The development of SRPK inhibitors has evolved through 3 stages: traditional ATP-site kinase inhibition (e.g., SRPINX31 and SRPIN340), specific inhibition of protein–protein interaction interfaces (e.g., DBS1), and covalent binding to target residues (e.g., C-DBS) [359]. Notably, SRPK inhibitors can shift VEGF splicing toward the VEGF165b variant (anti-angiogenic), thereby inhibiting tumor angiogenesis [360]. By modulating VEGF, SRPK inhibitors have entered clinical trials for diabetic macular edema, demonstrating therapeutic potential in reducing retinal vascular leakage [361]. SRPK1 inhibitors have shown potent anti-angiogenic activity in vitro by increasing VEGF165b levels, although their anti-tumor and anti-angiogenic effects in intrahepatic cholangiocarcinoma models have not yet been validated in vivo [362]. Overall, as the “activation switch” of SR proteins, SRPK represents a promising drug target for inhibiting tumor cell proliferation and neovascularization in bone-related malignancies.

In clinical and translational studies of bone-related diseases, we summarized validated AS-related cases. In PCa, detection of the AR-V7 variant in circulating tumor cells has been used to predict the efficacy of antiandrogen drugs [363]. This approach is particularly suitable for patients with bone metastatic CRPC, as bone is the most common metastatic site and often indicates therapeutic resistance [237]. In OA, chondrocytes re-express the COL2A1IIA variant in a “reverted” manner, and detection of COL2A1IIA-derived fragments in synovial fluid or blood, such as PIIANP, is commonly used to evaluate cartilage remodeling and disease activity [364]. PIIANP fragments have also been applied to assess cartilage damage in Kashin–Beck disease [365]. Soluble biomarkers based on COL2A1 AS variants can reflect OA progression earlier and with greater sensitivity than imaging examinations [101,366]. There are also representative cases in the field of AS-related therapies. Denosumab, used for the treatment of osteoporosis and giant cell tumor of bone, specifically recognizes and neutralizes certain sRANKL proteins generated by AS, thereby blocking the RANKL–RANK signaling pathway and efficiently suppressing osteoclast activity [367]. Finally, in bone defects or non-union fractures, miR-exon4 derived from exon 4 of AMELX promotes osteogenic differentiation by directly suppressing Nfia/Prkch and up-regulating RUNX2 in osteoblasts [69,368]. Leveraging advances in miRNA therapeutic delivery, the AS-derived miR-exon4 represents a compelling candidate for bone regeneration strategies, which requires further in vivo validation.

Small-molecule inhibitors targeting AS factors have encountered frequent clinical failure primarily due to off-target toxicities and limited tissue specificity, emphasizing the urgent need for strategies that enhance selective delivery in the context of bone tumor therapy. SF3B1 inhibitors such as E7107 were discontinued following cases of irreversible vision loss, while H3B-8800, despite alleviating transfusion dependence in a subset of MDS patients, did not achieve clearly defined clinical responses. PRMT5 inhibitors demonstrated efficacy in synthetic lethality models but raised safety concerns because of their broad roles in DNA repair and bone development. SRPK inhibitors can modulate VEGF AS and suppress angiogenesis, but their in vivo validation remains limited. To surmount these limitations, the development of next-generation strategies that achieve greater precision and tissue selectivity is imperative. Potential strategies include the design of nanocarriers responsive to the bone tumor microenvironment to achieve active targeting and minimize normal tissue exposure. The application of PROTAC technology to selectively degrade AS factors may provide a more precise therapeutic modality. Moreover, computer-aided screening coupled with AI-based predictive modeling offers opportunities to optimize the structural and pharmacological properties of small-molecule inhibitors. Finally, localized sustained-release delivery following bone tumor resection and patient stratification guided by AS variant biomarkers to enrich for populations most likely to benefit with minimal toxicity represent promising directions for the refinement of AS-targeted therapeutic strategies.

Overall, AS has emerged as a highly promising translational research direction in the diagnosis and treatment of bone-related diseases (Table 6). From diagnostic and prognostic biomarkers such as AR-V7 and COL2A1IIA/PIIANP, to therapeutic molecules such as Denosumab and miR-exon4, and further to nucleic acid drugs and small-molecule inhibitors under development such as ASO, AS modulators, and PRMT5/SRPK inhibitors, current evidence indicates that AS research is steadily moving from “mechanistic exploration” toward “clinical implementation”. The therapeutic success of AS-targeting drugs in SMA provides a strong rationale to accelerate their clinical translation in bone tumors, OA, osteoporosis, and bone regeneration. Future studies should focus on precisely defining pathogenic AS events under disease contexts, achieving tissue- and cell type-specific targeted intervention, and utilizing novel delivery systems such as bone-targeted nanocarriers to enhance drug accumulation at lesion sites. Therefore, elucidating the pathogenic mechanisms of AS in bone diseases and actively promoting interdisciplinary collaboration will be critical pathways to achieve precision diagnosis and targeted therapy in the future.

**Table 6.** Applications, challenges, and opportunities of AS-targeted therapeutics in bone diseases

Strategy	Applications	Challenges	Opportunities
Antisense oligonucleotides (ASOs)	1. Nusinersen: Restores SMN2 function by promoting exon 7 inclusion. 2. PKM ASO: Induces a PKM2-to-PKM1 switch, suppressing glycolysis and cell proliferation.	1. Require injection and face delivery barriers, especially across the blood–brain barrier and to bone tissue. 2. Unstable in vivo, prone to nuclease degradation and immune activation.	1. Chemical modifications (e.g., cEt and phosphorothioate backbone) enhance ASO stability. 2. Bone-targeted nanocarriers improve local delivery efficiency. 3. Enable personalized therapy for bone diseases caused by gene mutations.
Small-molecule splicing modulators	1. Risdiplam: Oral small molecule that promotes SMN2 exon 7 inclusion. 2. Branaplam (SMA/Huntington’s): Modulates 5′ SS, discontinued due to safety concerns. 3. SF3B1 inhibitors (E7107, Sudemycins, and H3B-8800): Block spliceosome assembly, induce tumor apoptosis. 4. PRMT5 inhibitors: Suppress glycolysis and proliferation in bone tumors; regulate DNA repair, enable synthetic lethality with PARP inhibitors. 5. SRPK inhibitors: Modulate VEGF splicing to inhibit angiogenesis.	1. Poor tissue specificity, broad off-target effects, notable toxicity. 2. High clinical failure risk (e.g., E7107 vision loss; H3B-8800 lacked defined responses).	1. High-throughput screening and AI modeling to optimize drug design. 2. Systemic distribution may benefit patients with chronic bone diseases. 3. PROTACs or bone-targeted nanocarriers to enhance specificity.
CRISPR/Cas-based approaches	Potential to directly correct splicing mutations underlying hereditary bone diseases (e.g., COL1A1/2, PHEX, and TMEM38B)	1. Limited efficiency of delivery to bone and cartilage tissues. 2. Long-term safety and off-target effects remain unresolved. 3. No clinical evidence available in bone diseases.	1. Monogenic bone disorders (e.g., OI and XLH) may represent ideal initial targets. 2. Development of bone-targeted delivery systems is essential to enable effective Cas enzyme activity.

## Conclusion and Future Perspectives

Over the past 2 decades, our understanding of AS processes in human tissues and their functions in bone biology has greatly expanded. Core spliceosome components and key factors regulating AS, initially identified in yeast splicing systems, have gradually been observed to have functional homologs in human cells, providing crucial insights into the conservation and specificity of eukaryotic AS. AS is extensively involved in bone tissue development, cellular differentiation, matrix remodeling, and activation of critical signaling pathways. The differentiation processes of various cell types in mature bone tissue, such as osteoblasts and osteoclasts, highly depend on AS regulation of key genes, which finely tune cell fate determination and functional differentiation pathways, thereby maintaining bone homeostasis and regenerative capacity. Notably, bone tissue exhibits distinct AS patterns of calcium channel genes. AS events in the RANK–RANKL signaling pathway contribute to bone remodeling, highlighting the critical role of AS in the dynamic development of the musculoskeletal system. These findings not only deepen our understanding of AS regulatory mechanisms in bone biology but also offer new directions for investigating the pathogenesis of bone remodeling disorders and developing AS-based therapeutic strategies. This review systematically compares the roles of AS in bone biology, bone tumor progression, and bone metastasis, revealing both shared mechanisms and context-specific regulation. Beyond intrinsic cellular AS regulation, it also highlights the pivotal role of AS within the bone microenvironment. Furthermore, from a clinical perspective, it comprehensively summarizes therapeutic strategies targeting AS and proposes their potential directions in translational research, providing new insights for future precision diagnosis and therapy.

In bone tumors, the ratio of different AS variants of the same gene is closely correlated with poor prognosis, suggesting that AS variants serve as critical biomarkers for tumor progression. Moreover, AS is widely implicated in biological processes, such as tumor proliferation, migration, apoptosis, angiogenesis, DNA damage repair, EMT, microenvironment remodeling of bone metastases, and TGF-β signaling pathways. Notably, given the subtle differences between AS variants, which typically involve only a single exon or short sequence, conventional antibody-based detection methods often lack specificity. Consequently, most studies have relied on PCR-based approaches for detecting AS. The clinical application of AS-derived biomarkers currently faces 2 major bottlenecks: insufficient detection accuracy and difficulties in quantification. RT-qPCR is limited by primer specificity and amplicon length, making it difficult to distinguish highly similar or longer AS variants, often resulting in reduced sensitivity. Therefore, more advanced methods such as droplet digital (dd) PCR, long-read sequencing, or targeted RNA capture sequencing are needed to improve detection accuracy. AT-qPCR has been proposed for variant quantification, but it lacks validation and standardization. In the future, establishing stable internal reference systems will be essential to ensure reproducibility. Developing highly specific antibodies that target proteins encoded by different AS variants is a promising direction for future studies. Abnormal AS patterns in tumors have emerged as a frontier in cancer research. However, the mechanisms and functions of aberrant AS in bone tumors remain insufficiently explored, and related studies are still in their infancy. In particular, it is necessary to conduct large-scale clinical cohort studies to validate prognostically relevant AS variants in OS, ES, and CS, in order to further establish their clinical utility in diagnosis, molecular subtyping, and prognostic evaluation. Moreover, scRNA-seq and long-read sequencing will be instrumental in constructing comprehensive splicing landscapes of skeletal cells during development and disease, and establishing bone disease-specific “splicing code” databases.

We also recognize the clinical translational potential of AS-related therapeutic strategies. Currently, 3 major approaches have been developed for AS-targeted therapy: directly targeting aberrant AS variants, modulating cis-regulatory elements of pre-mRNA, and regulating the activity of AS factor proteins. Among them, ASOs, siRNAs, and gene-editing technologies offer high specificity but face challenges such as poor delivery efficiency, limited biological stability, and immunogenicity. In contrast, small molecules targeting AS factors demonstrate more favorable in vivo distribution and pharmacokinetics, though they carry the risk of splicing off-target effects. Finally, the development of precise and highly specific molecular detection strategies for AS variants constitutes a critical avenue for future research.

Clinically, the successful application of AS-targeted therapies such as Nusinersen and Risdiplam in treating SMA provides strong proof of concept for the feasibility of precise AS modulation. These cases highlight that the accurate identification of AS variants, well-defined mechanisms of splice site regulation, and optimization of drug selectivity are key determinants in future drug development. In bone tumors, AS targets such as PKM and VEGF have shown therapeutic potential through ASO-mediated metabolic reprogramming and anti-angiogenesis. As an upstream regulator of AS factors, PRMT5 inhibition exerts synthetic lethality in tumor cells harboring splicing mutations and modulates the splicing of DNA repair regulators like TIP60 and SUV4-20H2, showing synergistic efficacy when combined with PARP inhibitors. Importantly, PRMT5 is also involved in regulating the differentiation of bone cell lineages; its inhibition enhances osteogenesis while suppressing osteoclast and chondrocyte differentiation, indicating its dual role in shaping the tumor microenvironment. Therefore, future research should focus on systematically identifying functional AS events in bone tumors, elucidating the crosstalk between splicing regulation and metabolic, immune, and DNA repair pathways, and developing ASO drugs with splicing specificity as well as tissue-selective AS factor inhibitors, to advance precision AS-targeted therapies in the clinical management of bone tumors.

Future research on AS in bone-related diseases could prioritize several critical directions. These include the comprehensive identification of functional AS events to clarify their specific roles in bone development, remodeling, and disease progression; the delineation of AS regulatory networks to uncover the hierarchical organization and pathway crosstalk mediated by key splicing factors; the development of splicing-specific and tissue-selective therapeutic strategies, including small-molecule inhibitors and ASOs; and the large-scale clinical validation of prognostically relevant AS variants to establish their utility in diagnosis, molecular subtyping, and risk stratification of bone tumors. Advancing these areas will not only deepen our mechanistic insights into AS-mediated regulation in bone biology and malignancies but also accelerate the clinical translation of AS-targeted therapies.
